# Regulatory Whack-a-mole: A Rapid Scoping Review of Gambling Marketing Restrictions and their (in)Effectiveness

**DOI:** 10.1007/s40429-026-00793-5

**Published:** 2026-09-17

**Authors:** Jamie Torrance, Jessica Smith, Alex M. T. Russell, Simon Wright, Leon Y. Xiao, Elen Wyn-Davies, Conor Heath, Philip Newall

**Affiliations:** 1 School of Psychology, Swansea University, Singleton Campus, Swansea, SA28PP UK; 2 Experimental Gambling Research Laboratory, School of Health, Medical and Applied Sciences, Central Queensland University, Rockhampton, Australia; 3 School of Creative Media, City University of Hong Kong, Hong Kong, China; 4 School of Psychological Science, University of Bristol, Bristol, UK

**Keywords:** Gambling marketing, Advertising regulation, Public health, Consumer protection, Harm reduction, Scoping review

## Abstract

**Purpose of Review:**

Gambling marketing increases participation, spending, and harm. Marketing restrictions represent the best regulatory response. Decades of tobacco and alcohol marketing regulation demonstrate a recurring pattern in which each restriction triggers industry adaptation through, e.g., channel substitution, alibi marketing, and the exploitation of regulatory gaps. However, no review has systematically assessed gambling operator adaptations to marketing restrictions. This review examines the effectiveness of gambling marketing restrictions implemented globally between 2010 and 2026, in light of these potential adaptations.

**Recent Findings:**

Fifty records spanning 13 jurisdictions were synthesised across six gambling marketing restriction categories. (1) *Content*, (2) *Timing*, and (3) *Channel Restrictions* were consistently undermined by poor operator compliance, redistribution of advertising to less regulated platforms, and narrow regulatory scopes leaving the majority of marketing exposure unregulated. (4) *Inducement Restrictions* yielded the strongest behavioural evidence, with sustained reductions in customer acquisition and money gambled, though operators restrategised by redirecting promotional effort towards retaining existing customers. (5) *Platform-enforced measures* curtailed on-platform content but not external gambling website traffic or underage exposure. (6) *Comprehensive Bans* were circumvented through alibi marketing, transitional exemptions, and reallocation of spend to unregulated, operator-owned media channels.

**Summary:**

Regulatory displacement was evident across all six categories. Operators consistently migrated marketing activity to less regulated channels, formats, and strategies following each restriction, mirroring the established regulatory trajectories of tobacco and alcohol marketing. Effective gambling marketing regulation requires statutory, comprehensive, and future-proof frameworks rather than the voluntary, circumventable, and reactive approaches evaluated in this review.

**Supplementary Information:**

The online version contains supplementary material available at https://doi.org/10.1007/s40429-026-00793-5.

## Introduction

Exposure to gambling marketing is associated with a range of harms [1, 2]. These include increased gambling participation [3–5], increased spending [6], the placement of riskier or unplanned bets [7], the normalisation of gambling among underage audiences [8, 9], and relapse among individuals attempting to reduce or cease their gambling [10, 11]. The public’s awareness of these potential harms has increased in jurisdictions that are worst affected, such as Australia and the UK [12–14], which has led to voluntary industry-led initiatives such as the imposition of warning labels and marketing restrictions. However, these Industry-led initiatives have proven largely ineffective when tested [15, 16], with for example industry-branded harm prevention adverts actually increasing viewers’ urges to gamble, likely due to their use of operator branding and imagery that is consistent with ordinary gambling adverts [17]. Although emerging independently-designed interventions such as inoculation-based videos [18] and counter-industry messaging [19, 20] appear more promising in testing, these strategies cannot compete with the natural consequences of gambling operators’ immense marketing budgets (e.g., high frequency of advertising or use of celebrity endorsements). Restrictions and regulations therefore represent the most promising defence against gambling marketing harms [21].

However, research in other public health domains shows that regulators are often left playing a game of ‘whack-a-mole’, restricting one practice only for industry to deploy new tactics or to exploit regulatory loopholes. Tobacco marketing regulation followed a decades-long trajectory in which narrow channel-specific restrictions were circumvented through sponsorship, point-of-sale displays, and brand stretching [22–24]. Comprehensive frameworks such as the WHO Framework Convention on Tobacco Control eventually closed the most significant displacement pathways [25, 26]. Alcohol marketing has followed a comparable arc, with voluntary codes consistently producing poor compliance and industry-led ‘alibi marketing’ strategies enabling operators to maintain brand visibility through nominally non-promotional channels [27, 28]. In both cases, meaningful reductions in exposure were only achieved when regulation shifted from partial, voluntary, and reactive approaches to comprehensive, statutory, and future-proof ones. A parallel pattern can already be observed in online gambling product design, where regulations targeting specific structural characteristics, such as celebratory sound effects on losses-disguised-as-wins, have led to sounds which satisfy the letter but not the spirit of new regulations [29], and broader platform design interventions have consistently been outpaced by operator adaptation [30, 31]. Despite these precedents, it currently remains unclear whether gambling marketing restrictions are following the same trajectory.

Numerous jurisdictions have implemented varied approaches in recent years, though most have focused on advertising, which is only one component of the broader marketing mix that also encompasses product design, pricing, placement, and public relations [32, 33]. Since July 2023, the Netherlands has prohibited untargeted gambling advertising and instead permits targeted online advertising only where operators can demonstrate that at least 95% of the audience reached is aged 24 or over [34]. Conversely, Belgium and Italy have implemented near-total advertising restrictions [35]. Australia has pursued the most varied programme of reform, combining broadcast watershed restrictions, volume caps, inducement bans, blocking of illegal offshore operators, and restrictions on direct marketing to self-excluded consumers through the national BetStop register [36]. From 2027, further measures will require age-verified online advertising and ban stadium and uniform advertising, though exemptions for influencer and podcast content remain [37]. Restriction is also spreading beyond Europe and Australasia. In North America, Ontario’s regulator banned the use of athletes in gambling advertising and restricted celebrities likely to appeal to minors, from February 2024. In Asia, India’s Promotion and Regulation of Online Gaming Act 2025 prohibits any advertisement that directly or indirectly promotes online gambling. In South America, Brazil introduced measures in July 2026 prohibiting urgency cues, portrayals of betting as investment, and commentator recommendations during live sport. Most such measures nonetheless target defined vulnerable groups rather than reducing exposure across the whole population, and because evaluation evidence can only accumulate once restrictions are in force, the geography of the evidence base lags the geography of regulation itself.

Limited systematic evaluation of these measures nonetheless leaves policymakers unable to distinguish genuinely effective restrictions from those that merely redirect activity across these channels. This is particularly pressing for jurisdictions such as the UK, where the demonstrated link between marketing and harm [7, 38] has strengthened the case for adopting a public health approach to gambling marketing regulation. Such an approach would prioritise preventing harm at the population level rather than relying on reactive measures targeted at individuals already experiencing gambling-related harm [39].

The gambling marketing restrictions literature spans diverse jurisdictions, regulatory frameworks, methodologies, and outcome measures. This breadth is a potential strength, as converging findings across different regulatory contexts carry more weight than any single jurisdiction’s evidence alone. However, systematic synthesis in this area remains scarce. Previous reviews have compared legislative frameworks across European jurisdictions [40], examined gambling policies’ effects on young people [41], and identified political will rather than evidence as the central catalyst for restriction adoption [42]. Others have drawn attention to a marked asymmetry in the evidentiary standards applied to gambling marketing policy. The liberalisation of gambling marketing remains largely unchallenged, in part because the public are rarely consulted and industry has little incentive to object, while the introduction of restrictions faces demands for robust proof of effectiveness [43]. The UK is a case in point. Despite having the largest evidence base documenting the harms of gambling marketing, it maintains some of the fewest marketing restrictions among comparator countries [42]. Although warranted, these reviews have charted rather than evaluated restrictions, and in some cases have addressed gambling marketing as one component of a broader policy landscape rather than a primary focus. What is currently missing is a synthesis that draws this evidence together to assess which gambling marketing restriction designs produce measurable reductions in exposure, whether their effectiveness is maintained over time, and whether marketing activity is genuinely reduced or simply displaced to less regulated channels and formats.

We therefore aimed to conduct a rapid scoping review to systematically evaluate the effectiveness of gambling marketing restrictions implemented globally over the past 16 years. Specifically, the review’s preregistered objectives were to:

1. Identify which restriction mechanisms achieve measurable reductions in marketing exposure, and how operators exploit regulatory gaps to undermine them.
2. Examine how restriction effectiveness changes over time, including the enforcement frameworks that differentiate successful policies and the displacement effects through which restrictions may redirect rather than reduce exposure.
3. Map where robust evaluation data exists versus limited evidence contexts.

## Methods

A rapid scoping review was conducted following a preregistered protocol (OSF: https://osf.io/du34z/overview?view_only=31fbf459f8324e7db66e1d1e0144e109*).* A rapid scoping review was selected in preference to a full systematic review for two reasons. First, the question concerned the breadth of restriction types, jurisdictions, and evaluation approaches rather than the pooled effect of a single intervention, and so we anticipated the included evidence as being too heterogeneous for meta-analysis. Second, gambling marketing regulation is changing quickly, and evidence of this kind has limited value if it arrives after the decisions it is intended to inform.

For the purposes of this review, ‘marketing’ was operationalised as any promotional activity undertaken by or on behalf of gambling operators to advertise or encourage engagement with gambling products, brands, or services across all formats and channels. ‘Restrictions’ was operationalised as any statutory, regulatory, or voluntary measure limiting or prohibiting the placement, content, or structural features of such marketing.

Searches were performed between January and April 2026 across six databases: APA PsycINFO, Medline, Scopus, Web of Science Core Collection, ASSIA, and ProQuest Psychology. Grey literature was searched through Google Scholar (first 300 results), preprint repositories (OSF Preprints, PsyArXiv, SocArXiv, SSRN), and targeted searches of international regulatory and governmental websites (e.g., the UK Advertising Standards Authority, Australian Victorian Responsible Gambling Foundation, and the Canadian Alcohol and Gaming Commission of Ontario). Searches were restricted to English-language records, and to regulatory and governmental websites publishing in English, because the review team had no translation capacity within the rapid timeline. The search strategy combined three conceptual blocks using AND, covering gambling terms (Gambl*, Bet*, Casino*, Wager*), marketing terms (Commercial*, Advert*, Market*, Campaign*, Promot*, Endors*, Sponsor*, Brand*, Induce*), and restriction terms (restrict*, regulat*, ban*, prohibit*, limit*, control*, polic*, legislat*, decree*, standard*, code*). Proximity operators linked the restriction and marketing blocks, and relevant subject headings were incorporated where available. The strategy was refined and executed by a subject librarian (EW-D) with specialist expertise in systematic reviews (see Supplementary File 1). Citation chasing (i.e., the systematic identification of additional records through the references and citations of eligible records) was conducted via CitationChaser, and the reference lists of eligible records were also manually reviewed as detailed below.

**Fig. 1:**
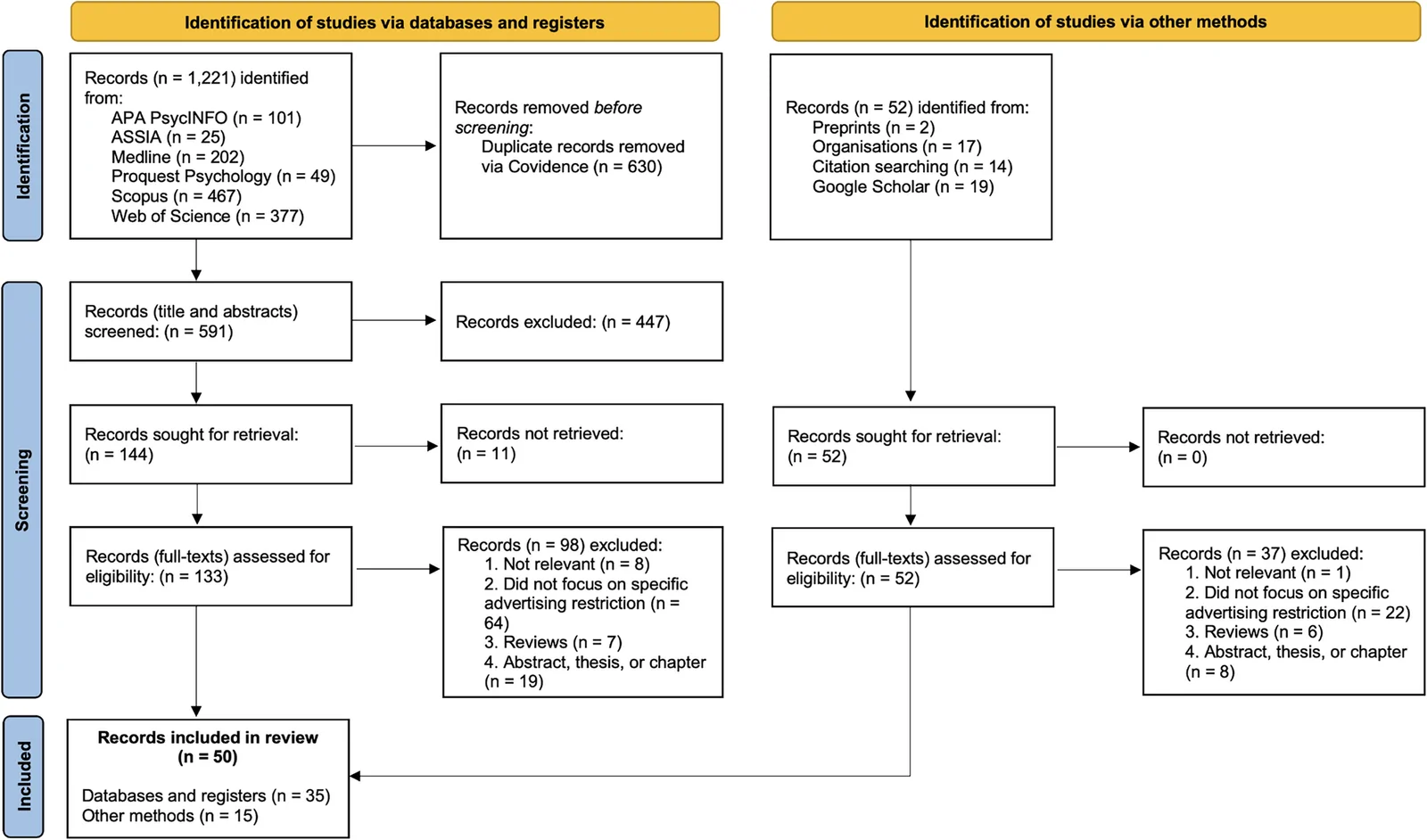
PRISMA flow diagram illustrating the identification, screening, and inclusion of records

English-language peer-reviewed studies, expert commentaries, and policy-relevant reports published between 2010 and 2026 were eligible provided they directly assessed, commented on, or related their findings to any specific gambling marketing restrictions. The 2010 start point was selected to capture the period of online gambling expansion, and the marketing practices the restrictions in this review target, consistent with the use of date limits in rapid review methodology. Records examining general advertising effects without reference to specific restrictions were excluded, as were systematic reviews, meta-analyses, conference abstracts, theses, and book chapters. All records were managed in Covidence. After removing duplicates, the titles and abstracts of the records (*n* = 591) were screened independently by two reviewers (JS and CH), yielding a Cohen’s κ of 0.82, indicating very good intercoder reliability [44]. Disagreements were resolved through discussions involving a third reviewer (JT) until full consensus (κ = 1.0) was reached. A further 52 records identified via other methods (preprints, organisational reports, citation searching, and Google Scholar) were incorporated at the full-text retrieval stage. The full texts of all records (*n* = 185) were screened by JS, CH, and JT, with 50% of the sample randomly selected for independent verification of screening decisions by a fourth reviewer (SW; κ = 1.0). Following exclusions (databases and registers, *n* = 98; other methods, *n* = 37), 50 records were included in the final review (databases and registers, *n* = 35; other methods, *n* = 15; see Fig. 1). The methodological quality of empirical studies was appraised using the Mixed Methods Appraisal Tool (MMAT; [45]. All empirical records met at least a good standard of methodological quality, and ratings were used to contextualise rather than determine inclusion.

Narrative synthesis was conducted to identify themes across the included records [46, 47]. Each policy document referenced within the included records was retrieved and reviewed by JT, CH, and JS, noting the nature of the restriction(s) imposed, the marketing activity targeted, and the underlying regulatory rationale. Restrictions sharing a common regulatory mechanism or target were then clustered to form candidate narrative ‘themes’ based on restriction typology, which were refined through regular discussion with the wider research team until a stable set was agreed.

## Results

**Fig. 2:**
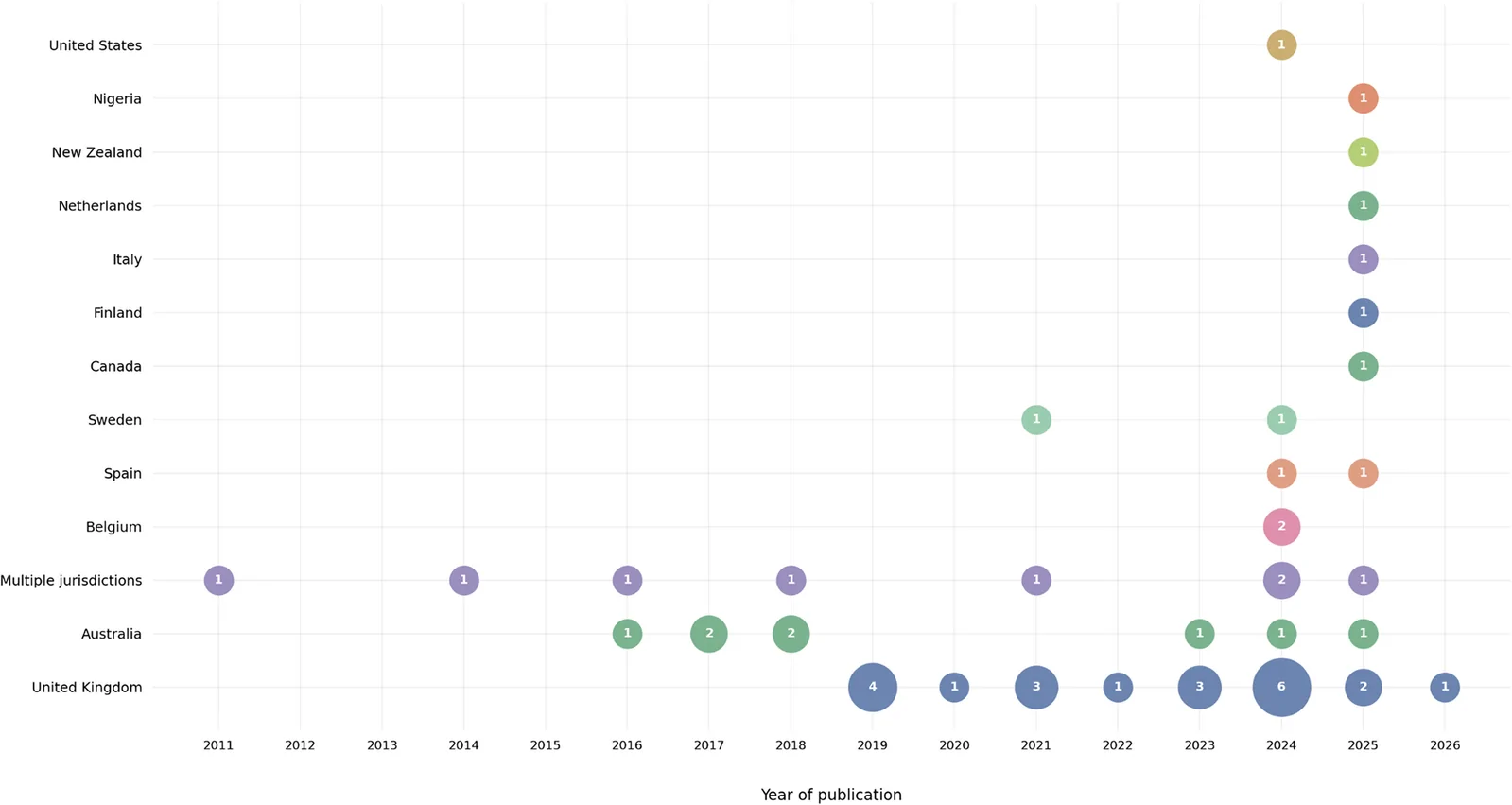
Distribution of included records (N = 50) on gambling marketing restrictions by jurisdiction and year of publication. Jurisdictions are ordered by total number of records then alphabetically (descending)

Fifty records met the inclusion criteria (see Table 1 for a summary of record characteristics, Fig. 2 for the distribution of records by jurisdiction and year of publication, and Supplementary File 2 for full study characteristics). Six categories of marketing restriction were examined, which provide the organising framework for the synthesis below: (1) content restrictions, (2) timing and scheduling restrictions, (3) placement and channel restrictions, (4) inducement and bonus restrictions, (5) platform and quasi-regulatory restrictions, and (6) near-total and comprehensive bans.

**Table 1:** Summary of included record characteristics (N = 50)

Characteristic	*N* (%)
Record type	
Empirical journal article	35 (70)
Technical report	9 (18)
Commentary or editorial	6 (12)
Empirical journal article design (*n* = 35)	
Quantitative	22 (62.9)
Mixed-methods	9 (25.7)
Qualitative	4 (11.4)

Percentages for record type are calculated from the full sample (N = 50). Percentages for empirical journal article design are calculated from that subsample (n = 35)

###  Content Restrictions

Content restrictions on gambling marketing, governing the portrayal, labelling, and targeting of promotional material, have been implemented across multiple jurisdictions but consistently fail to achieve meaningful compliance.

The earliest evidence emerged from Australia, where gambling advertising is regulated under the federal Interactive Gambling Act 2001, state- and territory-level gambling regulation legislation, and associated broadcasting and advertising codes of practice. A scan of 30 betting websites, conducted in 2016, found that inducement advertising rarely carried harm prevention messaging. When present, the messaging was often buried in complex terms and conditions [48]. A subsequent analysis of 280 television advertisements confirmed that incentive-based appeals dominated and inconsistent state-level warning guidance gave operators substantial latitude [49].

The UK evidence base is the most extensive on this topic and allows assessment of whether compliance has changed over time. Following the liberalisation of gambling advertising under the Gambling Act 2005, the regulatory architecture relies on the Committee of Advertising Practice (CAP) codes enforced by the Advertising Standards Authority (ASA), alongside the Industry Group for Responsible Gambling (IGRG) voluntary code, which has been criticised for outdated evidence, weak transparency, and limited accountability [50]. Early commentary noted that this framework, designed for broadcast, could not govern data-driven targeting, influencer content, or embedded digital formats [51]. Empirical work confirmed these concerns. Analyses of organic Twitter (now ‘X’) advertising found that 68 to 74% of gambling marketing breached at least one CAP requirement covering age restrictions, harm reduction, or identification as advertising [52, 53]. Consecutive analyses of Premier League broadcasts, news, radio, and social media in 2023 and 2024 found that only 14 to 18% of gambling references included harm reduction messaging and around 11% age restriction content. There was negligible change between measurement points despite the introduction of additional voluntary codes in the intervening period [54, 55]. The temporal comparison is particularly important for the question of whether self-regulatory frameworks can improve over successive iterations, and these findings suggest they have not.

Shortly before the 2018 FIFA World Cup, new UK regulatory guidance stated that advertising should not ‘unduly pressure the audience to gamble’. Nonetheless, a content analysis of all 64 matches found 69 live-odds advertisements across 32 ITV broadcasts, with a substantial share promoting complex markets difficult for consumers to evaluate and many featuring time or quantity-limited offers [56]. Newall et al. argued that many of these advertisements appeared inconsistent with the regulatory guidance’s aims.

Operators have also exploited two specific structural gaps in the UK framework. Content marketing, only belatedly recognised as advertising by the ASA, is significantly harder to identify as promotional than conventional advertising across all age groups. This essentially breaches CAP Code 2.1’s requirement that marketing be obviously identifiable [57], and represents a type of format displacement where restrictions on conventional advertising push operators towards less regulated promotional formats that may achieve equivalent or greater reach. CAP Code 16.3.12 prohibits gambling advertising of ‘strong appeal’ to under-18s, yet 19 of 24 social media adverts were found more appealing to those aged 11 to 24 than to adults aged 25 and over, with the 18 to 24 age group showing the highest appeal responses of any group [58]. Because the Code’s protection threshold is set at 18, the demographic most responsive to this form of marketing falls just outside its scope [59].

Swedish evidence shows that the precision of content restrictions matters more than the enforcement framework. The 2019 Gambling Act requires advertising to be conducted with ‘moderation’, but inconsistent interpretation of this term creates windows of opportunity for operators to position themselves at the limits of the law [60]. Campaigns have normalised gambling through gender-coded imagery and philanthropy framings while technically satisfying the standard [61]. Unlike the UK, Swedish evaluation data remain limited to qualitative case studies, making it difficult to assess whether compliance has shifted since the Act’s introduction.

Recent evidence from North American jurisdictions confirms the pattern extends across both statutory and self-regulatory frameworks. In 2018, the US Supreme Court ruled the Professional and Amateur Sports Protection Act (PASPA) unconstitutional, allowing individual states to legalise sports betting. Following the rapid expansion that ensued, around three-quarters of organic US gambling advertising on social media was non-compliant with the voluntary American Gaming Association (AGA) Code. Paid-for advertisements adhered to all assessed restrictions, suggesting that enforcement mechanisms attached to paid placement may be more effective than those governing organic content [62]. In Ontario, Canada, only 2.6% of approximately 4,000 gambling references across televised NBA and NHL matches carried harm reduction content under the Alcohol and Gaming Commission of Ontario (AGCO) framework [63]. As with the UK and Australian evidence, both North American contexts rely on single cross-sectional compliance assessments, meaning that neither the trajectory of effectiveness over time nor the behavioural consequences of non-compliance can be determined.

###  Timing and Scheduling Restrictions

Timing restrictions, including watershed bans (prohibitions during children’s viewing hours) and the UK’s voluntary whistle-to-whistle ban, consistently produced partial reductions in scheduled advertising windows but failed to curb overall exposure. Operators redistributed marketing to adjacent time slots, channels, and platforms outside each restriction’s scope. This pattern emerged under both statutory frameworks, as in Australia, and voluntary self-regulatory codes, as in the UK, suggesting that the enforcement mechanism alone did not account for the limitations of timing-based approaches.

Australian evidence provides early empirical support for these limitations. In March 2018, the Australian federal government prohibited gambling advertising during live sport broadcasts on television and radio between 5:00am and 8:30pm, from five minutes before the start of play until five minutes after its conclusion. Among 500 adolescents and adults surveyed around this period, 91% supported restrictions during children’s viewing hours, but 80% also endorsed a complete ban on gambling marketing during sports broadcasts, suggesting limited public confidence in timing-based measures alone [64]. Findings among 11 to 17-year-olds indicated that 67% reported exposure to gambling marketing before the 8:30pm threshold, 55% encountered it on social media platforms beyond the scope of the television watershed, and 84% continued to watch sport past the cut-off time. Moreover, 85% recalled at least one gambling brand, suggesting that substantial exposure persisted despite the watershed restriction [65, 66]. More recent analyses attribute this persistent exposure to marketing shifting into general programming and social media as on-air restrictions tightened [67, 68].

The UK evidence presents a comparable pattern. Prior to 2019, the Gambling Act 2005 permitted gambling advertising on television at any time of day, with no timing-based prohibition equivalent to those in other jurisdictions. Existing restrictions on marketing during children’s programming did not extend to sponsorship or branding by gambling operators [69]. During this period, 46% of young people and 71% of adults recalled gambling brands, with shirt sponsor recall particularly pronounced among adolescent boys who watched football [70].

Growing concern over this exposure led to the whistle-to-whistle ban. Introduced in August 2019 by the IGRG, the voluntary ban prohibited gambling operators from advertising on television from five minutes before the start of a live pre-watershed sporting event until five minutes after its conclusion. It did not extend to sponsorship, pitchside hoardings, or shirt branding. Pre-ban analysis confirmed that most gambling marketing already occurred in forms outside its scope, with 77% embedded within in-game coverage and 38% appearing on pitchside hoardings [71]. Post-implementation evaluations have shown only modest reductions during the restricted window, offset by increases in pre-match advertising and persistent in-game visibility through exempt channels [72, 73]. The Premier League analyses also cited in the content restrictions section found that gambling references accounted for 10.7% of content during restricted periods in 2023, rising to 39.5% of 23,690 references by 2024, suggesting that the restriction’s already limited impact may have diminished over time [54, 55]. The displacement effect was particularly visible during the 2022 FIFA World Cup, where 80.8% of gambling advertisements were concentrated in pre-match windows and 16% in post-match windows [74].

###  Placement and Channel Restrictions

Placement and channel restrictions regulate where gambling marketing may appear, covering sports sponsorship, social media, out-of-home advertising, and specific formats such as live-odds promotions. The evidence consistently shows that restrictions on specific channels leave the majority of gambling marketing exposure untouched, with operators substituting into adjacent channels beyond each restriction’s reach.

Australian evidence first established sponsorship as an under-regulated exposure mechanism [69], and more recent research found that young people reported continued exposure despite bans near schools and on public transport, describing current placement restrictions as outdated [68].

The UK evidence base on placement is the most developed. The brand recall and merchandising findings discussed in the timing Sect [70]., were extended by an analysis of child-facing football products, which found that 36 to 43% of Premier League trading cards featured visible gambling logos despite existing protections for children [75]. The voluntary Premier League front-of-shirt commitment, announced in 2023 and due to take effect from the 2026/27 season, has been subject to sustained scrutiny. Successive analyses of televised coverage have consistently shown that front-of-shirt logos account for only a small fraction of total gambling marketing visibility. Pitchside hoardings represented over half of gambling references, while shirt logos accounted for between 7% and 9% across studies [54, 55, 71]. A frequency analysis across 10 broadcasts from the 2022/23 season found that shirt-front gambling logos accounted for 6.9% of all gambling-associated logos. The voluntary ban will therefore apply to approximately 1 in 20 gambling logos visible during matches, and only 3.4% of all relevant logos featured harm-reduction or age-restriction warnings [76].

Social media represents the most significant enforcement blind spot in the UK placement landscape. As documented in the content restrictions section, betting advertising is widespread on platforms such as Twitter, is infrequently labelled, rarely incorporates age restrictions, and is accessible to substantial underage audiences [52, 53, 77]. Over three in five 11 to 17-year-olds had seen gambling advertising on online platforms, leading to arguments that platform design actively heightens exposure beyond what self-regulation can address [59]. Format-specific restrictions on live-odds and in-play advertising also remain under-developed. In-play betting accounts for around 50% of UK sports betting, but policy has focused on game integrity rather than advertising content [78].

At the local level, placement restrictions are inconsistently applied. In England, only 32% of 333 local authorities had any relevant commercial marketing policy on the placement of harmful marketing, including gambling [79]. In New Zealand, a study using wearable cameras with 168 children found that advertisements for self-regulated commodities including gambling were encountered more frequently than statutorily regulated ones such as tobacco [80].

###  Inducement Restrictions

Inducement restrictions, covering financial promotions, sign-up offers, free bets, and caps on wagering requirements, represent one of the most under-regulated areas of gambling marketing. Yet, the evidence base suggests that inducements are the form of marketing most directly linked to gambling behaviour. Restrictions in this area face distinctive challenges around disclosure, comprehension, and operator adaptation.

The Australian evidence discussed in the content restrictions section also established that inducements were poorly disclosed and rarely accompanied by harm prevention messaging [48]. Among 11 to 16-year-olds, inducement promotions were reported as the most influential form of gambling marketing encountered [65]. Australia subsequently introduced the National Consumer Protection Framework, which prohibits sign-up offers. However, a qualitative study of 20 young adults aged 18 to 24 found that almost half reported having used them, likely through offshore operators not subject to Australian regulations, and described the accessibility of promotions as a factor perpetuating their betting behaviour [81]. This represents a form of jurisdictional displacement distinct from the channel-level displacement seen in timing restrictions, with consumers accessing prohibited products through operators beyond the regulation’s reach.

Spain provides the strongest behavioural evidence. Royal Decree 958/2020 introduced restrictions on gambling marketing including a ban on using promotions to attract new customers and limits on bonus advertising and affiliate programmes. Previous research estimates that for every €1 operators invested in bonuses, gamblers deposited €1.60, and for every €1 in sponsorship, gamblers deposited €4, confirming the direct relationship between promotional spending and gambling behaviour. The Decree weakened most of these relationships, but notably the association between bonus spending and gambler deposits appeared to strengthen after the regulation took effect [6]. This suggests operators shifted promotional strategies away from customer acquisition and towards retention and reactivation of existing accounts. A longer-term time-series analysis of 2013 to 2023 data found that the Decree produced a permanent reduction in new accounts and total money bet, but had no effect on active accounts, deposits, or affiliate expenditure [82]. This is the clearest evidence in the current review that an inducement restriction can produce measurable behavioural change, but it also shows that operators adapted by redirecting effort towards channels and customer segments the regulation did not cover.

UK evidence is more recent and narrowly focused on wagering requirement comprehension. Ahead of the Gambling Commission’s 2026 cap on wagering requirements at a multiple of 10x, an experiment with 585 adult gamblers found that 92.4% underestimated the cost of a 10x requirement and only 5.5% estimated it correctly [83]. Disclosing the true requirement significantly reduced attractiveness, though higher-risk bettors appeared less deterred. Participants described inducements as manipulative and predatory. The prominence of inducements in the broader UK marketing landscape was confirmed by the World Cup analysis discussed in the timing restrictions section, where financial inducements accounted for 42.3% of gambling advertisements on ITV [74].

Within the UK, a Delphi study with 35 experts reached 93% consensus that price and discount promotions, bet-to-view arrangements, and urgency-inducing strategies should be banned [84]. However, as of August 2026, none of these measures have been introduced. More broadly, a review of 48 countries found that where inducement restrictions exist, they target specific vulnerable groups rather than population-level exposure, leaving promotional architectures largely untouched [85].

###  Platform and Quasi-Regulatory Restrictions

The compliance failures documented in broadcast and print contexts are mirrored, and in some cases amplified, in digital environments where platforms themselves increasingly operate as quasi-regulators. Platforms such as TikTok, Meta, and Google enforce licensing requirements, age verification, and harm prevention standards through mechanisms including account suspension and content moderation. These measures are not always aligned with national regulation.

Platform-level self-regulation can sharply reduce targeted on-platform content while leaving the underlying gambling activity largely untouched. An analysis of Twitch’s 2022 ban on unlicensed gambling streams found that it cut weekly gambling streams by 63.2% and total output by 44.3% among the streamers it targeted, with smaller spillover reductions even among streamers whose content was not directly banned [86]. Traffic to the banned gambling websites themselves showed no meaningful decline, however, and while the policy reduced gambling viewership among viewers classified as minors, it did so by slightly less than for other viewers [86]. Platform self-regulation thus curbed the visible content but failed to reduce either the gambling activity it was intended to address or fully reduce minors’ exposure to it.

Since 2013, Dutch law prohibits gambling advertising from targeting 18 to 23-year-olds. An analysis of advertisements published on Meta-owned social media platforms found reasonably high compliance among operators holding online gambling licences (92.7%) but a lower rate among those holding offline, land-based licences (70.2%) [34]. Where operators offered an explanation, they attributed the breaches to the default minimum targeting age in Meta’s automated advertising tools, which they had not overridden, suggesting that platform design can materially shape regulatory compliance. In Nigeria, by contrast, there are no specific statutory restrictions on gambling advertising, with the National Lottery Regulatory Commission instead relying on operator-adopted codes of practice. In that environment, a cross-sectional study of 406 undergraduates found near-universal exposure to sports betting advertising, and students who reported being influenced by it were more than four times as likely to experience significant gambling harms [87]. Taken together, these cases show the limits of relying on platform design or operator self-regulation in place of enforceable statutory restriction. In the Netherlands, a binding rule still left compliance gaps shaped by platform defaults, while in Nigeria, the absence of any specific advertising restriction left exposure pervasive and strongly associated with youth harm.

###  Near-Total and Comprehensive bans

Comprehensive or near-total bans represent the most restrictive regulatory endpoint and provide a natural counterfactual against which the partial restrictions considered in the preceding sections can be compared. A small number of European jurisdictions have pursued this approach, most notably Italy from 2019 and Belgium from 2023. Norway and Finland are sometimes grouped alongside these cases, but neither has prohibited gambling advertising in general. Both operate state monopolies in which marketing by any other operator is already prohibited, and both restrict the marketing of specific higher-risk products such as slot machines and casino games. The evidence below is drawn primarily from commentary and policy analysis, with behavioural data only beginning to emerge.

A consistent finding is that bans described as ‘total’ are, in practice, partial. Italy’s Dignity Decree 2019 was the first near-total European ban on gambling advertising. Early analysis anticipated that the measure would not slow market growth [88], and subsequent research confirmed this concern. Italian operators developed alibi brands such as ‘LeoVegas.news’ that mimicked gambling branding while technically avoiding direct promotion, maintaining high visibility in professional football despite the legal prohibition [89]. The ban also opened space for gambling-adjacent industries, with crypto and trading-app sponsorships moving into Italian and Spanish football to fill the gap left by gambling restrictions [76, 90]. This raises questions about whether comprehensive bans on gambling advertising simply redirect commercial pressure towards similarly risky products.

Belgium’s experience following the Royal Decree of July 2023 shows a similar pattern. Although widely described as a general ban, the regime contains several exceptions and transition periods. Sports sponsorships were permitted until 2024, limited shirt sponsorships until 2027, and amateur sports exemptions extend to 2028. Operators have exploited remaining legal channels, reallocating spend to their own media platforms, corporate social responsibility initiatives, podcasts, and live-streamed sports [35, 91]. A comparative analysis across seven jurisdictions noted that Italy’s ban has been politically contested on employment grounds and that the Netherlands has faced pressure to reverse its 2024 motion to ban online advertising for high-risk products, illustrating the political fragility of comprehensive restrictions even where they have been adopted [42].

Where population-level data exist, the evidence is mixed. An analysis across 30 European jurisdictions found that more restrictions on online gambling advertising were associated with lower rates of disordered gambling, though bans specifically did not show this association [92]. The Spanish behavioural data discussed in the inducement restrictions section showed effects on initiation (new accounts, total money bet) but not on continuation (active accounts, deposits), consistent with comprehensive restrictions being more effective at preventing new uptake than at changing existing behaviour [6, 82]. A Finnish survey of 132 active gamblers found that exposure remained widespread despite regulatory restrictions, with 60.5% wanting stricter regulation or a complete ban [93].

## Discussion

This review aimed to systematically evaluate the effectiveness of gambling marketing restrictions implemented globally over the past 16 years. Across 50 included records spanning six categories of restriction, the findings highlight that regulatory ambition was consistently undermined by operator adaptation and structural gaps in enforcement. This pattern mirrors the well-documented regulatory trajectories of tobacco and alcohol marketing.

**Fig. 3:**
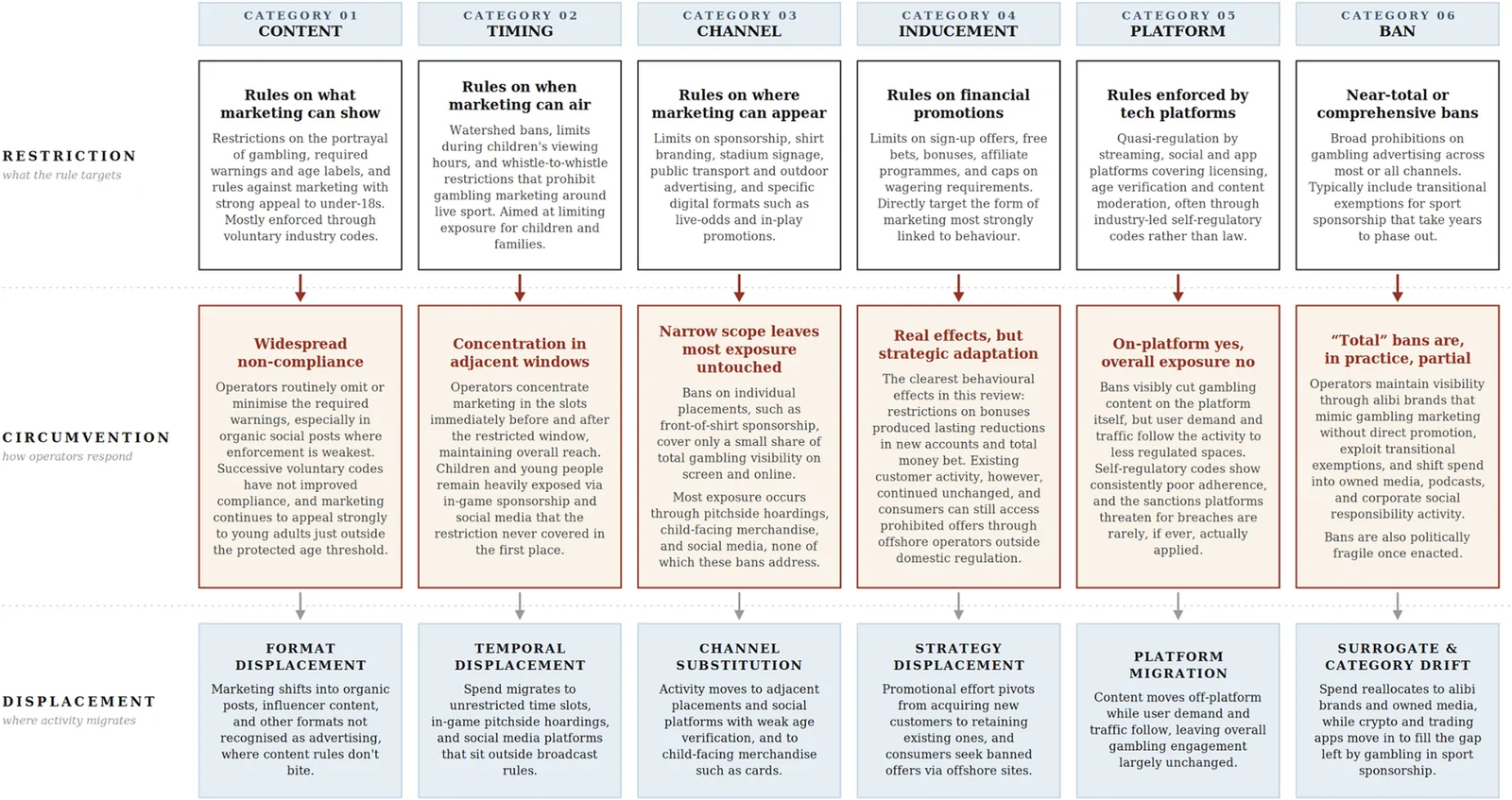
How gambling marketing restrictions are circumvented across six regulatory categories

Content restrictions showed persistently poor compliance under both statutory and self-regulatory frameworks, with UK evidence revealing negligible improvement across successive voluntary codes [50, 70]. Timing and scheduling restrictions, including Australia’s broadcast watershed and the UK’s whistle-to-whistle ban, produced partial reductions in scheduled advertising but triggered redistribution to pre-match slots, social media, and exempt formats such as pitchside hoardings [50, 69, 72, 73]. Placement and channel restrictions left the majority of exposure unregulated, with for example front-of-shirt bans applying to a small fraction of total visibility and social media remaining a largely unaddressed pathway [54, 55, 76]. Inducement restrictions produced the clearest behavioural effects, particularly in Spain where Royal Decree 958/2020 reduced new account creation and total money bet, though operators adapted by redirecting promotional effort towards customer retention rather than acquisition [6, 82]. However, parts of the Decree have since been struck down by the Spanish Supreme Court, and whether these reductions are maintained remains to be seen [82]. Platform-level measures such as Twitch’s ban on unlicensed gambling streams reduced on-platform content but failed to diminish traffic to gambling websites [86]. Even near-total bans in Italy and Belgium proved porous, with operators exploiting transitional exemptions, developing surrogate branding strategies, and reallocating spend to operator-owned media channels [89]. Taken together, these findings substantiate the regulatory ‘whack-a-mole’ dynamic in which each restriction suppresses marketing activity in one domain only for operators to rapidly redirect effort into adjacent, less regulated spaces (see Fig. 3 for a summary of this restriction, operator circumvention, and displacement dynamic).

Beyond this displacement dynamic, the varied magnitude of these effects warrants interpretive caution. The size of any measured effect is largely a function of which restriction is being evaluated rather than whether marketing restriction works in principle. Inducement restrictions act on a transactional mechanism, which is why the Spanish evidence detected reductions in new account creation and money bet within a defined window [6, 82]. Removing a single placement such as front-of-shirt sponsorship is different, stripping out one component of an otherwise saturated environment in which exposure persists across broadcast, social media, and owned channels. A small or undetectable change in harm may not definitively indicate that restriction is ineffective, only that narrow, single-channel measures are likely the wrong unit of intervention. Spain illustrates this directly. Royal Decree 958/2020 includes timing restrictions alongside its inducement measures, but the available literature has focused almost exclusively on the inducement side [82], leaving the timing component unevaluated. This is the central case for comprehensive restriction. Isolating the harm attributable to any individual marketing element is inherently difficult when harm is multiply determined and the wider ecosystem remains intact [73], and demands for such element-specific proof, frequently invoked to resist regulation, set an evidentiary bar that no comparable domain of public health regulation has been required to clear [43].

The findings of this review highlight how gambling marketing restrictions are following the same protracted regulatory trajectory as tobacco and alcohol marketing. Operators have responded to each new restriction through channel substitution, alibi marketing, and exploitation of regulatory gaps, precisely the strategies that characterised earlier stages of tobacco and alcohol control. However, gambling may be even more susceptible to this dynamic than its public health predecessors. The digital delivery of gambling products means that marketing, product design, and platform architecture are deeply intertwined, enabling operators to adapt at a speed and scale that was not possible with physical commodities like tobacco and alcohol [31, 94]. The difference is that the evidentiary groundwork already exists. Whether policymakers repeat this decades-long cycle or break it will depend on whether the lessons of tobacco and alcohol regulation are treated as directly applicable rather than loosely analogous.

The included literature reinforces this position. A large number of records called for the replacement of self-regulatory frameworks with statutory, independently enforced legislation [35, 42, 71, 82], alongside comprehensive restrictions spanning all marketing channels rather than targeting individual placements in isolation [50, 62, 64, 70, 74]. Australia’s parliamentary inquiry into online gambling offers a notable example. Informed by 161 submissions and 13 public hearings, the inquiry concluded that partial bans on gambling advertising do not work and recommended a comprehensive programme of reform spanning advertising, sponsorship, inducements, and platform regulation [95]. The protection of children and young people featured prominently in both the parliamentary inquiry and the broader included literature here, encompassing proposals to extend the regulatory definition of ‘young people’ to age 24 [58], ban content marketing that circumvents identifiability requirements [57, 77], and develop social media-specific regulations with improved age verification [52, 53, 68, 86]. Stronger regulation of inducements was also widely advocated, including disclosure requirements modelled on financial product regulation [48, 83]. Underpinning many of these proposals was a broader shift towards treating gambling marketing as a commercial determinant of health at the population level rather than an issue of individual responsibility [6, 50, 85], coupled with calls for ongoing compliance monitoring capable of adapting to operator strategies as they evolve [60, 89]. The convergence of these recommendations across diverse jurisdictions and methodologies reinforces the conclusion that the direction of reform is well established, even if its implementation lags behind.

The UK evidence base features prominently in this review. This partly reflects the earlier liberalisation of its gambling market and the sustained research effort that followed. However, it is also a function of the review’s English-language scope, which favours jurisdictions where relevant research is published in English. The patterns documented in the UK are already emerging in jurisdictions that liberalised gambling more recently, with early US compliance data following the 2018 Supreme Court decision showing familiar trends [62] and Canadian provinces, such as Ontario, generating initial evidence of widespread non-compliance under their new iGaming frameworks [63]. Critically, the evidence assembled in this review only exists because independent research groups chose to systematically document regulatory outcomes. In the absence of such work, the evidential default available to policymakers is that voluntary codes are functioning as intended. Research evidence is, of course, only one input into gambling policy. It is weighed against industry lobbying, fiscal interests, and political priorities, and political will rather than evidence has been identified as the central catalyst for restriction adoption [42]. Independent evaluation therefore does not determine policy outcomes, but its absence removes the only systematic counterweight to industry claims of self-regulatory success. Restrictions are typically introduced with little advance notice, limiting the scope for prospective evaluation designs. However, the field is well positioned to respond to this challenge. The compliance monitoring methodologies, exposure measurement frameworks, and displacement tracking approaches represented across the records in this review collectively form a toolkit that researchers in newly liberalised jurisdictions can adopt and adapt. Policymakers can also support this effort by mandating operator data disclosure as a condition of licensing, giving researchers access to the behavioural and transactional data needed to assess regulatory impact [96, 97]. The evidence base to inform effective restriction design now exists across multiple jurisdictions and restriction types. Translating that evidence into practice will depend on whether the research community can mobilise coordinated evaluation efforts at the speed required to keep pace with operator adaptation.

Several limitations of this review should be acknowledged. First, only English-language records were included, which may have excluded relevant research from non-Anglophone jurisdictions, though the review’s coverage of Spain, Italy, Belgium, the Netherlands, Sweden, and Finland through English-language publications suggests that the major European policy developments were captured. A related and broader gap concerns the jurisdictions represented. All 13 are high-income countries, predominantly in Western Europe, North America, and Australasia. No evidence was identified from Central or Eastern Europe, and Nigeria was the only record from the global South. This is unlikely to be an artefact of the language restriction alone, as evaluations of gambling marketing restrictions appear largely absent in these settings. Regulatory frameworks, market structures, and the political conditions shaping restriction adoption differ substantially across these contexts, so the displacement dynamics documented here should not be assumed to generalise to them. Second, the MMAT was used as a relatively brief quality assessment instrument, though methodological quality was used to contextualise rather than determine inclusion, consistent with scoping review aims where breadth takes precedence over hierarchical quality filtering. Third, the rapid timeline constrained the depth of searching and data extraction compared with a full systematic review, though the use of six databases, grey literature searching, and citation chasing provided a comprehensive foundation spanning 13 jurisdictions across 16 years of policy activity.

Several forthcoming developments will provide opportunities to test the conclusions drawn here. Finland’s partial transition to a licensing system for online casino and betting from July 2027 will offer a natural experiment, with Norway’s continued monopoly model serving as a comparator. Italy and Belgium remain the clearest existing test cases of near-total bans, but neither has yet produced the outcome data needed to determine whether comprehensive restrictions reduce gambling-related harm or primarily redirect operator activity into channels the ban does not reach. Prospective evaluation of these transitions, designed in advance of implementation, would substantially strengthen the evidence base. Comparative designs applying common outcome measures across jurisdictions would be particularly valuable, as the predominantly cross-sectional, single-jurisdiction evidence available here cannot establish whether broader restriction packages are circumvented more or less readily than narrow ones, or whether operator adaptation is more pronounced in some regulatory contexts than others. Answering either question would require measurement approaches consistent enough that differences between jurisdictions reflect operator behaviour rather than differences in study design.

## Conclusions

Gambling marketing restrictions, as currently implemented, have been consistently undermined by operator adaptation. Each restriction has prompted operators to redirect effort to channels, formats, and strategies beyond that restriction’s reach. This dynamic was evident across all six restriction categories, every enforcement framework, and all 13 jurisdictions represented in the evidence base. The trajectory closely parallels that of tobacco and alcohol marketing regulation, where decades of piecemeal, reactive measures preceded the comprehensive statutory frameworks that eventually proved more effective. Gambling regulation needs not repeat this cycle. The independent research community has built a substantial and growing evidence base capable of informing anticipatory, comprehensive, and statutory approaches, and continued research efforts will ensure it keeps pace with operator adaptation. The gap, however, is no longer primarily in the evidence, but in the political will to act on it.

## Key References

**García-Pérez Á, Krotter A, Aonso-Diego G. The impact of gambling advertising and marketing on online gambling behavior: an analysis based on Spanish data. Public Health. 2024;234:170–7. https://doi.org/10.1016/j.puhe.2024.06.025. A time-series analysis of whether Spain's 2020 advertising restrictions changed online gambling behaviour and industry marketing spend.
**De Jans S, Hudders L, Newall P. Gambling advertising still exists in Belgium despite a widely reported ‘ban’. Addiction. 2024;119:1324–6. https://doi.org/10.1111/add.16458. A commentary showing Belgium's 2023 advertising ‘ban’ is full of exemptions, so gambling adverts persist in various forms.
*Heath C, Torrance J, Xiao L, Newall P. Loopholes and logos: How ‘decoy brands’ perpetuate gambling advertising in Italian football. J Gambl Issues. 2025; https://doi.org/10.4309/MLHD3076. Documents how operators use decoy/alibi brands and logos to keep gambling visible in Italian football despite advertising restrictions.
*Rossi R, Nairn A. Clearly (not) identifiable – The recognisability of gambling content marketing. Int J Mark Res. SAGE Publications; 2025;67:33–52. https://doi.org/10.1177/14707853241292953. An experiment finding children largely fail to recognise gambling content marketing as advertising.
*Torrance J, Wright S, Newall P, Crawford T, Quigley M, Dymond S. (Mis)Comprehension and (Mistaken) Attractiveness of Financial Gambling Inducements among UK Bettors. J Gambl Stud. 2026; https://doi.org/10.1007/s10899-026-10491-6An experiment showing UK bettors overwhelmingly miscomprehend the true cost of free bet inducements, even under the 2026 x10 wagering cap.

## Supplementary Information

Below is the link to the electronic supplementary material.

Supplementary Material 1

Supplementary Material 2

## Data Availability

No datasets were generated or analysed during the current study.
