# Supplementary material for: Regulatory Whack-a-mole: A Rapid Scoping Review of Gambling Marketing Restrictions and their (in)Effectiveness
Source: Curr Addict Rep. 2026 Sep 17;13(1):85. doi: 10.1007/s40429-026-00793-5 (PMC13585857; doi:10.1007/s40429-026-00793-5)
Supplement: Supplementary file 1 [file 40429_2026_793_MOESM1_ESM.pdf]

Rapid scoping review of Gambling Marketing Restrictions.

Six electronic academic databases will be searched: Medline, APA PsycINFO, Web of Science, SCOPUS, ASSIA, and ProQuest Psychology. Prior to search strategy optimization, we anticipate that search terms will employ three conceptual blocks. The first block encompasses gambling terms such as: gambl* OR bet* OR wager*. The second block captures marketing concepts such as: market* OR advertis* OR promot* OR sponsor* OR brand* OR induce*. The third block identifies restriction terms such as: restrict* OR regulat* OR ban* OR prohibit* OR limit* OR control* OR polic* OR legislat* OR decree* OR standard* OR code*. Boolean 'AND' operators will connect these blocks to identify literature at the intersection of gambling AND marketing AND restrictions., and relevant organizational websites such as the Advertising Standards Agency, Victorian Responsible Gambling Foundation, and Gambling Commission. Finally, reference lists of all eligible studies will be manually reviewed to identify any relevant records that may have been missed through the database and grey literature searches.

To supplement the database searches, grey literature including preprints, organizational reports, and technical documents will be identified through Google Scholar (first 300 results), alongside targeted searches of repositories including OSF preprints, PSY/SOCArxiv, SSRN (conducted up to April 1st 2026)

Grey Literature - (e.g. Advertising Standards Agency (ASA), Victorian Responsible Gambling Foundation, Gambling Commission) will be searched

Scoping search run 21st January 2026
Library Officer: Elen Wyn Davies

Inclusion	Exclusion
2010-current	Pre-2010
All study types	
Any Population	
English language	
Journal articles	
Grey literature	

Banning	Adverts	gambling
Restrictions	Marketing	Betting
Regulations	Campaign	Casino

Prohibiting	Promotion	Poker
Limiting	Brands	
Control	Induce	
Policies	Sponsor	
Legislation		
Decree		
Standards		
Code		

Search strategy
APA PsycINFO (Via Ebscohost)
Search run 26th January 2026 Filters applied 2010-current ; English

1. XB(restrict* OR regulat* OR ban OR bans OR banning OR prohibit* OR limit* OR control* OR polic* OR legislat* OR decree* OR standard* OR code*) N1 (Commercial OR Commercials OR Advert* OR Market* OR Campaign* OR Promot* OR Endors* OR Sponsor* OR Brand* OR Induce*)
2. XB(Gambl* OR Bet OR Bets OR Betting* OR Casino* OR Wager* OR Poker)
3. DE "Gambling"
4. #2 OR #3
5. #1 AND #4

Search strategy
Scopus (Via Elsevier)
Search run 26th January 2026 Filters applied 2010-current ; English

1. TITLE-ABS-KEY (restrict* OR regulat* OR ban OR bans OR banning OR prohibit* OR limit* OR control* OR polic* OR legislat* OR decree* OR standard* OR code*) W/1 (Commercial OR Commercials OR Advert* OR Market* OR Campaign* OR Promot* OR Endors* OR Sponsor* OR Brand* OR Induce*)
2. TITLE-ABS-KEY (Gambl* OR Bet OR Bets OR Betting* OR Casino* OR Wager* OR Poker)
3. #1 AND #2

Search strategy
Medline (Via Ebscohost)
Search run 26th January 2026 Filters applied 2010-current ; English

1. XB(restrict* OR regulat* OR ban OR bans OR banning OR prohibit* OR limit* OR control* OR polic* OR legislat* OR decree* OR standard* OR code*) N1 (Commercial OR Commercials OR Advert* OR Market* OR Campaign* OR Promot* OR Endors* OR Sponsor* OR Brand* OR Induce*)
2. XB(Gambl* OR Bet OR Bets OR Betting* OR Casino* OR Wager* OR Poker)
3. MH “Gambling”
4. #2 OR #3
5. #1 AND #4

Search strategy
Web of Science Core Collection (Via Clarivate)
Search run 26th January 2026 Filters applied 2010-current ; English

1. TITLE-ABS-KEY (restrict* OR regulat* OR ban OR bans OR banning OR prohibit* OR limit* OR control* OR polic* OR legislat* OR decree* OR standard* OR code*) NEAR/1 (Commercial OR Commercials OR Advert* OR Market* OR Campaign* OR Promot* OR Endors* OR Sponsor* OR Brand* OR Induce*)
2. TITLE-ABS-KEY (Gambl* OR Bet OR Bets OR Betting* OR Casino* OR Wager* OR Poker)
3. #1 AND #2

Search strategy
ASSIA (Via Proquest)
Search run 26th January 2026 Filters applied 2010-current ; English

1. title((restrict* OR regulat* OR ban OR bans OR banning OR prohibit* OR limit* OR control* OR polic* OR legislat* OR decree* OR standard* OR code*) N/1 (Commercial OR Commercials OR Advert* OR Market* OR Campaign* OR Promot* OR Endors* OR Sponsor* OR Brand* OR Induce*)) OR abstract((restrict* OR regulat* OR ban OR bans OR banning OR prohibit* OR limit* OR control* OR polic* OR legislat* OR decree* OR standard* OR code*) N/1 (Commercial OR Commercials OR Advert* OR Market* OR Campaign* OR Promot* OR Endors* OR Sponsor* OR Brand* OR Induce*))
2. title(Gambl* OR Bet OR Bets OR Betting* OR Casino* OR Wager* OR Poker) OR abstract(Gambl* OR Bet OR Bets OR Betting* OR Casino* OR Wager* OR Poker)
3. MAINSUBJECT.EXACT("Gambling")
4. #2 OR #3
5. #1 AND #4

Search strategy
Proquest Psychology (Via Proquest)
Search run 26th January 2026 Filters applied 2010-current ; English

1. title((restrict* OR regulat* OR ban OR bans OR banning OR prohibit* OR limit* OR control* OR polic* OR legislat* OR decree* OR standard* OR code*) N/1 (Commercial OR Commercials OR Advert* OR Market* OR Campaign* OR Promot* OR Endors* OR Sponsor* OR Brand* OR Induce*)) OR abstract((restrict* OR regulat* OR ban OR bans OR banning OR prohibit* OR limit* OR control* OR polic* OR legislat* OR decree* OR standard* OR code*) N/1 (Commercial OR Commercials OR Advert* OR Market* OR Campaign* OR Promot* OR Endors* OR Sponsor* OR Brand* OR Induce*))
2. title(Gambl* OR Bet OR Bets OR Betting* OR Casino* OR Wager* OR Poker) OR abstract(Gambl* OR Bet OR Bets OR Betting* OR Casino* OR Wager* OR Poker)
3. MAINSUBJECT.EXACT("Gambling")
4. #2 OR #3
5. #1 AND #4

Seed Papers:

Aonso-Diego, G., García-Pérez, Á., & Krotter, A. (2025). Impact of Spanish gambling regulations on online gambling behavior and marketing strategies. *Harm Reduction Journal*, *22*(1), Article 107. https://doi.org/10.1186/s12954-025-01219-7 (Not in PsycINFO) (In SCOPUS)

Wilson, J., Rossi, R., Bransden, N., Amos, M., & Sakis, P. (2024). Drivers of Gambling Marketing Restrictions–An International Comparison: University of Bristol & Ipsos.

McGrane, E., Pryce, R., Wilson, L., Field, M., & Goyder, E. (2025). How did the 'whistle-to-whistle' ban affect gambling advertising on TV? A live football matching study. *Addiction Research & Theory*, *33*(2), 134–142. https://doi.org/10.1080/16066359.2024.2355183 (PsycINFO)

García-Pérez, Á., Krotter, A., & Aonso-Diego, G. (2024). The impact of gambling advertising and marketing on online gambling behavior: an analysis based on Spanish data. *Public Health (London)*, *234*, 170–177. https://doi.org/10.1016/j.puhe.2024.06.025 (PsycINFO) (SCOPUS)

Constandt, B., & De Jans, S. (2024). Insights into the Belgian gambling advertising ban: the need for a comprehensive public policy approach. *Health Promotion International*, *39*(4). https://doi.org/10.1093/heapro/daae116 (Not in PsycINFO) (In SCOPUS)

Citation Chasing of Seed Papers in CitationChaser
