# Supplementary material for: Regulatory Whack-a-mole: A Rapid Scoping Review of Gambling Marketing Restrictions and their (in)Effectiveness
Source: Curr Addict Rep. 2026 Sep 17;13(1):85. doi: 10.1007/s40429-026-00793-5 (PMC13585857; doi:10.1007/s40429-026-00793-5)
Supplement: Supplementary file 2 [file 40429_2026_793_MOESM2_ESM.pdf]

## Supplementary File 2. Characteristics of included records (N = 50)

MMAT = Mixed Methods Appraisal Tool quality rating; N/A = not applicable (non-empirical records).

| Study | Authors | Year | Setting | DOI (for studies) URL (for reports) | Record type | MMAT | Study design | Advertising types examined / Marketing channels | Restriction/ policy examined | Restriction/ policy document(s) | Findings/outcomes | Restriction/policy implications | Policy recommendations | Knowledge gaps (future research) |
|---|---|---|---|---|---|---|---|---|---|---|---|---|---|---|
| Aonso-Diego et al., 2025 | Alonso-Diego, G., García-Pérez, Á., & Krotter, A. | 2025 | Spain | https://doi.org/10.1186/s12954-025-01219-7 | Empirical Study | ***** | Quantitative time-series analysis using secondary data from regulatory reports | Advertising expenditures, Bonus expenditures, Sponsorship expenditures, Affiliation expenditures of gambling operators | Royal Decree 958/2020. | • Spanish Royal Decree (https://www.boe.es/eli/es/rd/2020/11/03/958/dof/cat/pdf) | Between 2013–2023, marketing expenditure increased substantially, including an 83.23% rise in advertising spend, a 449.30% increase in bonuses (from €30m to €166m), a 259% rise in affiliate spending, and a 130.84% increase in sponsorship spending. Following the introduction of the Spanish Royal Decree, there was a sustained reduction in gambling activity, particularly in the number of new accounts and total money wagered from Q2 2021 onwards, compared with prior trends. The regulation also led to reductions in operator spending on advertising, bonuses, and sponsorship. However, it did not impact the number of active accounts or deposits made, and affiliate programme expenditure remained unaffected. | It suggests that regulatory measures affect non-gamblers, reducing the likelihood of initiating gambling behaviour, but do not influence active gamblers. Additionally, although the legislative measure covers a large part of the marketing strategies of gambling operators, the gambling industry continues to advertise in different ways. Gambling operators can advertise on their channels (e.g., websites, social media profiles) and exploit the Internet advertising legal loophole. Despite restrictions on affiliate programmes (e.g., limiting hours, targeting minors, and including responsible gambling labels), no changes were observed in this marketing channel. This may reflect the challenges for regulators in controlling the content and reach of numerous, diverse affiliate accounts. | Researchers highlight the importance of drawing lessons from other addictive behaviours to inform gambling regulation. Effective legal regulations should be prioritised, with ongoing monitoring and adaptation as industry strategies evolve, rather than relying primarily on self-regulation. | Further research is needed to assess whether the effects on online gambling behaviour persist long-term, particularly following the Spanish Supreme Court ruling 527/2024, which nullified several key articles of Royal Decree 958/2020. |
| Constandt & De Jans, 2024 | Constandt, B., & De Jans, S. | 2024 | Belgium | https://doi.org/10.1093/heapro/daae116 | Editorial | N/A | N/A | Gambling advertising generally | Article 61, Royal Decree 2023, Determining the conditions for advertising games of chance, In text referred to as Royal Decree July 2023 | • Belgium Royal Decree (https://www.ejustice.just.fgov.be/mopdf/2023/03/08_3.pdf#Page2) | Although Belgium's decree is described as a "total ban" on gambling advertising, it is more accurately a partial ban with several exceptions and transition periods. Sports sponsorships are still permitted until 2024, with limited shirt sponsorship allowed until 2027 and amateur sports exemptions extending to 2028. Additional exceptions include business-to-business advertising, incidental broadcast exposure, venue-based promotions, and certain online advertising through rights holders. As a result, gambling companies are increasingly exploiting remaining legal avenues, reallocating budgets to their own media channels, supporting sports-related charitable causes, and targeting amateur sports. The common industry argument that a total or partial advertising ban would push consumers to | In Belgium, enforcement of the Royal Decree on gambling advertising is inconsistent due to an understaffed and under-resourced gambling authority, limiting the ban's effectiveness. Additionally, Belgium's partial ban on gambling advertising, with multiple exceptions and transition periods, has allowed companies to exploit remaining legal avenues, highlighting the need for more comprehensive and enforceable restrictions. | To be effective, a total ban on gambling advertising is both appropriate and necessary to denormalise gambling, particularly among children, and to disrupt pathways to gambling products and brands. A strong, independent regulatory body is essential to ensure that such a ban is meaningful rather than merely symbolic. Belgium's experience demonstrates that a partial ban alone is insufficient to prevent gambling-related harm and provides valuable lessons for other countries considering advertising restrictions. | Ideally, gambling policies should be based on independent scientific evidence, free from influence by the gambling industry or other commercial interests. However, further data is needed to fully evaluate the impact of specific policy measures. |

| Study | Authors | Year | Setting | DOI (for studies) URL (for reports) | Record type | MMAT | Study design | Advertising types examined / Marketing channels | Restriction/ policy examined | Restriction/ policy document(s) | Findings/outcomes | Restriction/policy implications | Policy recommendations | Knowledge gaps (future research) |
|---|---|---|---|---|---|---|---|---|---|---|---|---|---|---|
| | | | | | | | | | | | unregulated and unsafe gambling markets is not supported by scientific research. | | | |
| Daniel et al., 2025 | Daniel, F. M., Essien, E. A., Ukoaka, B. M., Emeruwa, V. E., Charlie, A. M., Udam, N. G., Kelechi, V. K., & Ameh, S. | 2025 | Nigeria | https://doi.org/10.1007/s44192-025-00214-5 | Empirical Study | **** | Quantitative cross-sectional analytical study using self-administered questionnaire with undergraduate students (N = 406) | The influence of gambling advertisement | The National Lottery Regulatory Commission (NLRC) codes of practice. | N/A | This study showed that 36.7% undergraduate students had engaged in sports betting and 30.3% had gambled in the past year. Male students, particularly those aged 18–25 and from higher socioeconomic classes, were more likely to gamble, favouring football and online platforms, with regular gambling occurring 2–3 days per week. Problem gambling was prevalent (42.7% of past-year gamblers) and negatively affected academic activities. Motivations included winning money, earning income, and having fun, while exposure to advertisements influenced gambling behaviour and predicted problem gambling. | Participants who reported seeing promotional sports betting advertisements and acknowledged that these ads often motivated them to place bets were more likely to develop gambling disorders. The lack of strict regulations has left young people particularly vulnerable to aggressive marketing during sports events across multiple media channels. | To reduce exposure to harmful gambling products and prevent problem gambling, marketing should prioritise individual well-being. While the NLRC encourages responsible gambling measures such as age verification, self-exclusion, and deposit limits, this approach has been criticised for shifting responsibility onto individuals rather than the industry. More robust regulation is proposed, including bans on betting promotions during live-streamed fixtures, restrictions on advertising near schools and universities, and tighter controls on social media marketing for users under 30, identified as a higher-risk group for gambling susceptibility. | Future research should examine the long-term effects of preventive measures, the social., psychological factors regarding gambling, and advertising influences on sports bettors. A comprehensive approach that combines education, regulation, community engagement, and mental health support can help create a safer environment and promote responsible financial habits among students who engage in sports betting. |
| De Jans et al., 2024 | De Jans, S., Hudders, L., & Newall, P. | 2024 | Belgium | https://doi.org/10.1111/add.16458 | Commentary | N/A | N/A | Gambling advertising generally | Ban on gambling advertisement referred to as Article 61, Royal Decree 2023, Determining the conditions for advertising games of chance. | • Belgium Royal Decree (https://www.ejustice.just.fgov.be/mopdf/2023/03/08_3.pdf#Page2) | Although Belgium's new gambling advertising regulation is often described as a general ban, it permits several forms of advertising, including operator-owned websites, social media, search engine ads targeting interested consumers, and sponsorship of amateur sports clubs. Transitional arrangements restrict most sports stadium sponsorship from 2025 and shirt sponsorships from 2028, while lottery products remain exempt. Operators are also using corporate social responsibility initiatives, podcasts, prize draws, and live-streamed sports to maintain visibility, exploiting legal loopholes similar to strategies previously used by the tobacco industry. | In response to the new regulation, gambling operators are exploiting loopholes and alternative strategies to maintain brand visibility. While much public gambling advertising has disappeared, a complete ban has not yet been implemented, so advertising continues to exist in Belgium | N/A | N/A |
| Díaz et al., 2024 | González Díaz, K., Cisneros Örnberg, J., & Reitan, T. | 2024 | Sweden | https://doi.org/10.1080/14459795.2024.2323044 | Empirical Study | **** | Qualitative study with semi-structured interviews ( N = 12), seminars and press conferences, as well as policy documents and | Gambling advertising generally | Gambling Act 2019 | • Unofficial English translation Gambling Act 2019 Swedan (https://www.spelinspektionen.se/globalassets/dokument/engelska/gambling-act-sfs-2018_1138-uppdate-sfs- | All stakeholders recognised that the law regulates advertising content rather than volume, but interpretations of "moderation" varied. Industry and media actors acknowledged a shared responsibility to ensure moderation, though they reported difficulties in applying rules consistently, particularly in relation to high-risk products and bonus promotions. Views on regulation did differ, some | The conceptual confusion may reflect gambling companies' inclination to position themselves exactly within the limits of the law or to challenge these. Legislation with ambiguous definitions can open a "window of opportunity" for stakeholder negotiation and incremental interpretation. The | Generally, legislation needs to be accurate and clearly defined, yet flexible enough to be applicable in different situations. Ambiguous definitions in legal concepts can sometimes increase the likelihood of policy adoption. They also suggest that rapid changes to newly introduced regulations | Investigations of how legislation concerning gambling advertising and public health varies between countries, and how policy processes and the choice of policy instruments are constructed and implemented, are obvious venues for |

| Study | Authors | Year | Setting | DOI (for studies) URL (for reports) | Record type | MMAT | Study design | Advertising types examined / Marketing channels | Restriction/ policy examined | Restriction/ policy document(s) | Findings/outcomes | Restriction/policy implications | Policy recommendations | Knowledge gaps (future research) |
|---|---|---|---|---|---|---|---|---|---|---|---|---|---|---|
| | | | | | | | statements analysed using framework analysis. | | | 2024_255.pdf) • Original document (https://svenskforfattningssamling.se/sites/default/files/sfs/2018-06/SFS2018-1138.pdf) | supported stricter laws, others preferred self-regulation or allowing market consolidation. A clear divide emerged between government, which prioritised public health and consumer protection, and industry, which emphasised commercial freedom and downplayed advertising's role in harm. While stricter measures (including potential bans) were widely discussed, support organisations expressed low confidence in operators' compliance with regulation. | Swedish case demonstrates that policy-making is interactive and ongoing, with stakeholders actively shaping how legal concepts are applied in practice | may be premature, as the full effects are not apparent until the initial regulations have had time to take effect. | future research. |
| Di Censo et al., 2025 | Di Censo, G., Delfabbro, P., & King, D. L. | 2025 | Australia | https://doi.org/10.1007/s11469-025-01457-7 | Empirical Study | ***** | Qualitative online survey with 18-24 year olds ( N = 20) with thematic analysis | Gambling advertising generally and specifically sign-up offers | National consumer protection framework for online wagering in Australia – National policy statement (restricting sign-up offers) | • Australia Online Wagering consumer protection framework (https://www.dss.gov.au/system/files/resources/national-policy-statement-updated-3-may-2022.pdf) | The main themes identified were initial interest, perpetuating factors, and perceptions of risk. Young Australians often developed an initial interest in sports betting through early exposure via family and peers, advertising that normalised betting, and personal motivations such as the desire to win money or try a new hobby. Their betting behaviour was maintained by a fear of missing out on potential winnings, shared participation with peers, and the accessibility of promotions. Participants were also aware of the potential negative consequences of disordered gambling, including financial., psychological., and interpersonal harms. Additionally, participants observed that advertising primarily showcased male actors and utilised male endorsers and voiceovers. | Despite Australia prohibiting signup offers almost half (n=9) of the participants reported using them. | These findings indicate that policy interventions aimed at reducing existing social norms and restricting advertising could potentially reduce interest in sports betting and mitigate gambling harm. | N/A |
| Djohari et al., 2019 | Djohari, N., Weston, G., Cassidy, R., Wemyss, M., & Thomas, S. | 2019 | UK | https://doi.org/10.1186/s12954-019-0291-9 | Empirical Study | **** | Mixed methods involving quantitative data and interviews analysed with thematic analysis with 71 families. Consisting of young people (8-16) ( N = 99) and adults ( N = 71) | Sponsorships and Sports betting advertisement | ASA codes on content restrictions for under 18s & Watershed Ban | • CAP Codes for Under 18s (https://www.asa.org.uk/static/d9dd9d06-00e7-4630-81d460b598c7d976/gambling-and-lotteries-advertising-protecting-under-18s.pdf) • Watershed Ban (https://bettingandgamingcouncil.com/uploads/22703-BGC-IGRG-Code-7th-Edition-111023.pdf) | Just under half of young people (46%) and over two thirds of adults (71%) could name at least one gambling brand unprompted, with higher recall among boys and those who frequently watched football. Around two thirds of young people (63%) correctly matched at least one shirt sponsor to a football team, and 30% matched three or more. Among adults, 62% correctly matched at least one sponsor. Young people most commonly recalled gambling advertising via television, digital screens, and sport-related contexts, while adults reported exposure through television, digital screens, stadium advertising, and betting venues. Overall, gambling was widely perceived as normalised in sport, reported | These findings question the effectiveness of existing regulations, including the 9pm watershed and exemptions allowing sports betting advertising during live broadcasts. Although a ban on advertising during live sport has been widely proposed in the UK, the results suggest this alone would have limited impact on reducing young people's exposure. Despite restrictions prohibiting gambling advertising in youth-focused media since 2007, substantial proportions of young people still reported exposure, particularly via screens, digital platforms, and | Policymakers need to take a comprehensive approach, similar to strategies used in tobacco control, that considers all forms of gambling advertising, including promotions and sponsorships. They should also evaluate how recent changes in Australia have succeeded, or fallen short, in reducing young people's exposure to gambling marketing. The study emphasises the urgent need for robust regulation that addresses the variety of channels and tactics the gambling industry uses to promote its products. | Given the limited research currently available in the UK, policymakers may need to draw on evidence from Australia to guide policy and public health prevention responses. The similarity of findings across contexts suggests that applying a principle of 'logic based on parallel evidence' could be a useful interim approach until UK-specific research is produced. |

| Study | Authors | Year | Setting | DOI (for studies) URL (for reports) | Record type | MMAT | Study design | Advertising types examined / Marketing channels | Restriction/ policy examined | Restriction/ policy document(s) | Findings/outcomes | Restriction/policy implications | Policy recommendations | Knowledge gaps (future research) |
|---|---|---|---|---|---|---|---|---|---|---|---|---|---|---|
| | | | | | | | | | | | by 78% of young people and 86% of adults. | social media. This suggests that regulating only television advertising (e.g. commercial breaks) is insufficient to meaningfully limit exposure among younger audiences. | | |
| Djohari et al., 2021 | Djohari, N., Weston, G., Cassidy, R., & Kulas-Reid, V. | 2021 | UK | https://doi.org/10.1080/14660970.2020.1860028 | Empirical study | ***** | Quantitative, observational content analysis design, using children's most common forms of engagement with football: 1. sticker albums; 2. trading cards; and 3. football magazines to quantify visibility of gambling sponsorship | Sponsorships and media advertisement (in relation to child exposure) | Guidelines from the Advertising Standards Agency, Committee of Advertising Practice and Broadcast Committee of Advertising Practice gambling rules relating to the protection of children | • CAP Code (https://www.asa.org.uk/static/6e35da0a-c47f-4ab8-9767e4e7635bfec9/16-gambling-cap-code.pdf)<br>• BCAP Code (https://www.asa.org.uk/static/699c12ab-3a81-4175-9a22f8b900997394/6f1bc1a1-6d6c-4e63-9990fc0a4ee766cf/The-BCAP-Code-Gambling.pdf)<br>• AP/BCAP Guidance: "Protecting Children and Young People" (https://www.asa.org.uk/static/uploaded/734c1499-850d-4d2f-88a441ffd1903b50.pdf) | Of the 607 official trading cards available for the Match Attax Premiership 2017/18 season, 220 cards (36.24%) featured a visible gambling logo. For the Panini Adrenalyn XL Premiership 2019/20 season, 212 cards (42.57%) out of 498 contained gambling logos. Gambling logos were visible across all magazines. They found logos for 20 different gambling brands: Betway, 32Red, SportPesa, M88/Mansion, OPE, TLC Bet, 888 Sport, LaBa360, Bet Victor, Fun88, W88, ManBetX, Dafabet, LeoVegas, SkyBet, Party Casino, Bet365, Betdaq and Granada Bingo (shirt sponsors<br>at the time of Coventry City's FA Cup win in 1987). | Despite legislation and self-regulation in the UK intending to reduce the exposure of children and young people to gambling advertising. The research shows how mobile these logos are within a visual economy. The stickers and magazines aimed at adults can be refracted multiple times into the football-related worlds of child fans. | The authors argue for tighter restrictions on how, where and when gambling is advertised, particularly in association with sports that are popular with children. Legislation and self-regulation intended to reduce the exposure of children to gambling advertisements and branding should also consider the nature of children's engagement with football as well as the presence of gambling logos in products that are marketed to, and consumed by, children. Policies need to take into account the varied contexts to which children are exposed to football-related media. | The widespread presence of gambling logos within media predominantly aimed at children highlights the need for greater scrutiny of how such content is reproduced and disseminated within child-oriented spaces. Future research could improve consistency by standardising logo measurement and explore whether publishers deliberately obscure gambling logos in children's media. |
| GambleAware, 2024 | Gamble Aware | 2024 | UK | https://www.gambleaware.org/our-research/publication-library/articles/gambling-marketing-in-great-britain-what-needs-to-change-and-why/ | Commentary | N/A | N/A | Gambling advertising generally | Gambling Act (2005), GRG Code for Socially Responsible Advertising, Watershed ban, whistle-to-whistle ban, Premier League commitment to ban front-of-shirt logos, UK Code of Broadcast Advertising. | • Gambling Act 2005 (https://www.legislation.gov.uk/ukpga/2005/19/pdfs/ukpga_20050019_en.pdf)<br>• GRG Code, watershed (https://bettingandgamingcouncil.com/uploads/22703-BGC-IGRG-Code-7th-Edition-111023.pdf)<br>• Whistle-to-Whistle (https://www.gov.uk/government/publications/high-stakes-gambling-reform-for-the-digital-age/high-stakes-gambling- | There is strong evidence in the current research that gambling marketing is widely seen by children and young people, contributing to the normalisation of gambling and increasing the risk of harm. Advertising is also linked to greater harm among individuals already experiencing problem gambling. Public concern about the volume and impact of gambling advertising is high, yet current health messaging and signposting to support are limited and inconsistent. There is strong public support for increased gambling marketing regulation. | The over-reliance on self-regulatory mechanisms has proven ineffective at ensuring best practice. Voluntary agreements, such as the whistle-to-whistle ban, have been found to be tokenistic. The latest (7th78) edition of the IGRG Code for Socially Responsible Advertising has a number of issues, including poor maintenance (e.g., broken links), use of stigmatising language, and reliance on outdated evidence. It lacks transparency around marketing spend, enforcement actions, and the evidence underpinning campaigns, and fails to promote accountability or the sharing of best practices. | There needs to be an approach to reduce gambling harm that focuses on commercial determinants of health at a whole-population level. The paper recommends 3 strategies: (1) A pre-watershed ban13 on all broadcast gambling advertising (TV/Video On Demand/Radio) including broadcast sponsorship; (2) All gambling marketing to include independent evidence-led health warnings with effective signposting to support; (3) A ban on gambling marketing at sports events (including removal of sponsorships from sports clothing, merchandise, and wider stadium). | N/A |

| Study | Authors | Year | Setting | DOI (for studies) URL (for reports) | Record type | MMAT | Study design | Advertising types examined / Marketing channels | Restriction/ policy examined | Restriction/ policy document(s) | Findings/outcomes | Restriction/policy implications | Policy recommendations | Knowledge gaps (future research) |
|---|---|---|---|---|---|---|---|---|---|---|---|---|---|---|
| | | | | | | | | | | reform-for-the-digital-age)<br>• Premier league commitment- (https://resources.premierleague.pulselive.com/premierleague/document/2025/08/08/38e43722-3117-4a04-9755-dac24052b85d/PL-Gambling-Code-of-Conduct-07.08.25.pdf)<br>• BCAP code (https://www.asa.org.uk/static/40293b82-443a-4416-9555ff5259585538/8ae45746-492a-4c9c-91ee1d4fb018f6f2/The-BCAP-Code.pdf) | | | | |
| García-Pérez et al., 2024 | García-Pérez, Á., Krotter, A., & Aonso-Diego, G. | 2024 | Spain | https://doi.org/10.1016/j.puhe.2024.06.025 | Empirical study | ***** | Quantitative longitudinal study using a Seasonal Autoregressive Integrated Moving Average (SARIMA) model | Gambling advertising generally, specifically Incentives, promotions and sponsorships | Spanish Royal Decree 958/2020 | • Spanish Royal Decree (https://www.boe.es/eli/es/rd/2020/11/03/958/dof/cat/pdf) | Findings show that investment in advertising promotions, and sponsorships significantly increase the number of new and active accounts, deposits, and total money bet. It has been estimated that, for every €1 invested in bonuses and sponsorship, gamblers deposit €1.6 and €4 into their accounts, respectively. The Spanish RD regulating advertising in gambling appeared to mitigate the relationship between marketing expenditure and gambling behaviour, with the exception of expenditure on bonuses, which seems to have strengthened. | The Spanish law did reduce the link between general marketing strategies The use of bonuses still strongly influences gambling behaviour, making it a key area for regulators to monitor and people's gambling activity, but didn't eliminate it entirely. Although spending on bonuses isn't statistically proven to increase new accounts under the law, the confidence interval suggests there might still be some effect, it's just weaker than before. This matters because the law completely bans using promotions to attract new clients. Since attracting new gamblers with promotions is banned, gambling operators may have shifted their focus to keeping existing players active or bringing back inactive ones | These results underline the importance of ongoing monitoring and regulation of gambling behaviour in Spain, emphasising the need for strict adherence to regulations. Effective legislation can go beyond limiting marketing, it can include measures like fewer operator licences, Limits on money bet, prizes, and losses, raising the minimum gambling age, and increasing penalties for breaking the rules. The results of this study underscore the importance of adopting a public health perspective | Other countries may look to Spain's example in monitoring gambling data and accessing it. Additionally, future research should evaluate the impact of regulatory actions on gambling behaviour over time following their implementation.. |
| Han et al., 2025 | Han, Q., Yang, J., & Simonov, A. | 2025 | Across jurisdictions (Research conducted in China) | http://dx.doi.org/10.2139/ssrn.5298054 | Empirical study | **** | Quantitative-High-frequency panel analysis of Twitch livestreaming data covering | Livestreaming Gambling | Twitch ban on unlicensed gambling livestreams September 20, 2022 | • Twitch Regulations (https://www.twitch.tv/p/safety/education-portal/en/articles/gambling-education/) | Twitch's October 2022 ban on unlicensed gambling streams substantially reduced gambling content, with banned streamers (streamers who have streamed banned content) cutting gambling content by 63% and overall streams by 44%, and | The banning policy reduced gambling livestreams and caused spillover effects on unbanned content and streams in other languages. Reductions in minors' viewership and | The analysis underscores the importance of protecting minors from potentially harmful content. The study call's for complementary actions that aim to protect | Future research should use broader streamer datasets and explore policy effects across platforms and competitive dynamics. Additionally, studies should investigate how |

| Study | Authors | Year | Setting | DOI (for studies) URL (for reports) | Record type | MMAT | Study design | Advertising types examined / Marketing channels | Restriction/ policy examined | Restriction/ policy document(s) | Findings/outcomes | Restriction/policy implications | Policy recommendations | Knowledge gaps (future research) |
|---|---|---|---|---|---|---|---|---|---|---|---|---|---|---|
| | | | | | | | the top 6,000 live streamers | | | | unbanned streamers also showing smaller reductions, indicating spillover effects. The policy had stronger effects on popular streamers, extended to streams in other languages. On the demand side, total hours watched and engagement declined, particularly among casual viewers, while loyal viewers remained engaged. For viewers likely to be minors, exposure to gambling streams decreased, though slightly less than for adults. However, the ban had little effect on traffic to gambling websites, suggesting it primarily shaped behaviour on Twitch rather than online gambling overall. | engagement were smaller, indicating the policy was slightly less effective for protecting them. The findings highlight the value of self-regulation on digital platforms, rather than blanket bans, platforms can design policies with narrower scopes but still induce broad behavioural changes by leveraging creator heterogeneity and motivation. | minors, such as identifying potential minor users and downranking regulated content in their recommendations, to strengthen minor protection. | creators respond to policy interventions and whether findings generalise to other content regulations, as well as examine whether platform regulations drive qualitative changes in content, such as increased reliance on loyal users or AI-assisted production. |
| Heath et al., 2025 | Heath, C., Torrance, J., Xiao, L. Y., & Newall, P. | 2025 | Italy | https://doi.org/10.4309/MLHD3076 | Commentary, Letter to the editor | N/A | N/A | Decoy brands | Belgium Royal decree, referred to in text as Belgium and Italian Communications Authority Resolution No.132/19/CONS | • Belgium Royal Decree (https://www.ejustice.just.fgov.be/mopdf/2023/03/08_3.pdf#Page2)<br>• Italy Communications Authority (https://www.agcom.it/sites/default/files/migration/delibera/Delibera%20132-19-CONS.pdf) | An oversaturation of gambling advertising in professional sports is a common feature in jurisdictions that have liberalised gambling. Even in countries with stricter regulation, such as Belgium and Italy, loopholes and brand substitution strategies allow operators to continue high levels of promotion. They highlight how gambling advertising in Italian football exploits legal loopholes through "decoy brands" that mimic gambling operators' branding while technically avoiding direct promotion of gambling. | Decoy brands undermines the intent of Italian advertising restrictions, maintaining high visibility of gambling brands despite the law. | Italian policymakers should be aware of potential loopholes that the gambling industry may look to exploit to undermine legislation aimed to protect gamblers from harm. It is important to monitor industry compliance in jurisdictions with those legal restrictions, as even small loopholes might be exploited. | N/A |
| Hetherington & Phillips, 2023 | Hetherington, B., & Phillips, T. | 2023 | Australia | https://responsiblegambling.vic.gov.au/resources/publications/discussion-paper-gambling-harm-and-the-online-gambling-environment-1145/ | Discussion paper | N/A | N/A | Gambling advertising generally | Broadcasting Services Act 1992, referred to in text as 'current restrictions on gambling advertising that apply to a narrow range of children's TV hours and live sport between 5 am and 8.30 pm' & National Consumer Protection Framework | • Broadcasting service Act (https://www.legislation.gov.au/C2004A04401/latest/downloads)<br>• National Consumer Protection framework (https://www.dss.gov.au/system/files/resources/national-policy-statement-updated-3-may-2022.pdf) | Online gambling increases harm by allowing continuous, instant, and remote betting. Market pressures and technology encourage risky products and spending, with inducements and easy credit compounding the problem. Young people increasingly see gambling as a normal part of sport, and underage esports betting with skins can normalise gambling, create unrealistic expectations, and cause harms similar to adult gambling. | Recent studies show that sports betting harms are rising despite policy measures, such as limited advertising restrictions during children's TV and early live sports. New forms of gambling, like loot boxes and in-game wagering, largely fall outside legal definitions and have minimal regulation. Efforts to limit advertising, such as Australia's 2018 restrictions on live sport, have simply shifted gambling ads into general programming rather than reducing their overall presence. | Interventions to reduce the risks associated with simulated gambling and gambling-like products in video game content warrant consideration. Strengthened restrictions on direct marketing and inducements would reduce the risk of gambling harm | N/A |
| Hing et al., 2016 | Hing, N., Sproston, K., Brook, K., & Brading, R. | 2016 | Australia | https://doi.org/10.1007/s10899-016-9642-6 | Empirical Study | ***** | Quantitative content analysis, repeated measures, capturing the range and | Wagering Inducements | Commonwealth laws (including the Interactive Gambling Act 2001 and the Broadcasting Services Act | • Interactive gambling act 2001 (https://www.legislation.gov.au/C2004A00851/latest/download | A scan of 30 major race and sports betting websites identified 223 financial inducements across 15 inducement types, including sign-up bonuses, referral offers, free bets, and enhanced odds. These offers were heavily | The study indicates that many structural features of sports and race betting inducements do not align with harm minimisation or consumer protection goals. Most inducements lack responsible gambling | The findings highlight the need for stronger, clearer regulation of wagering inducements to promote safe betting. Regulations should define responsible gambling objectives, clarify which | People experiencing significant gambling harms* report harmful effects of wagering inducements, but more robust empirical research with larger samples is needed, |

| Study | Authors | Year | Setting | DOI (for studies) URL (for reports) | Record type | MMAT | Study design | Advertising types examined / Marketing channels | Restriction/ policy examined | Restriction/ policy document(s) | Findings/outcomes | Restriction/policy implications | Policy recommendations | Knowledge gaps (future research) |
|---|---|---|---|---|---|---|---|---|---|---|---|---|---|---|
| | | | | | | | structural features of inducements offered | | 1992); State laws (e.g., Gambling Regulation Act 2003 Victoria); mandatory codes of practice (e.g., Northern Territory Code of Practice for Responsible Gambling); voluntary codes of practice (e.g., Queensland Responsible Gambling Code of Practice); individual operators' codes (e.g., Tabcorp Wagering Responsible Gambling Code of Conduct); and broadcasting and advertising codes of practice. | s)<br>• Broadcasting services Act 1992 (https://www.legislation.gov.au/C2004A04401/latest/downloads)<br>• Gambling regulation Act 2003 Victoria (https://content.legislation.vic.gov.au/sites/default/files/2025-10/03-114aa110-authorised.pdf)<br>• Northern Terrirtory Code of Practice (https://nt.gov.au/_media/docs/business-and-industry/gambling-and-racing/gambling-codes-of-practice/nt-code-of-practice-responsible-gambling-2022.pdf)<br>• Queensland Responsible Gambling (https://www.publications.qld.gov.au/dataset/202496ee-ba88-479a-9c8e-7dc765133f21/resource/7c0d2522-e5eb-47a8-bd7b-41e0638c632e/download/qld-responsible-gambling-code-of-practice.pdf)<br>• Tabcorp Code of conduct (https://help.tab.com.au/troubleshooting-support/tabcorp-wagering-responsible-gambling-code-of-conduct)<br>• Broadcasting and advertising codes of practice; Radio (https://cra.au/hubfs/SIMON_E | promoted but buried in complex terms and conditions, making them hard to understand, particularly playthrough requirements.Very few inducement advertisements included responsible gambling messages, raising concerns about informed consumer choice. Additionally, The terms and conditions of many wagering inducements are complex, not always explicit in their advertisements, difficult to find, sometimes embedded in a raft of general terms and conditions, and obscured by legalistic language | messages, and their terms and conditions often fail to support informed choice. | inducements are acceptable, improve transparency of terms and conditions, mandate responsible gambling messages, and consider banning misleading offers linked to harm. Advertising of inducements, especially during times accessible to minors, should be reduced, and a consistent national approach with active government oversight is recommended. Enhanced monitoring, compliance, and complaints mechanisms are also necessary to ensure consumer protection and maintain public confidence. | particularly to assess differences across inducement types. Overall, there is a lack of evidence on sports and race betting inducements, limiting evidence-based policy. Key gaps include whether features like betting "happy hours" encourage binge gambling and whether inducements prompt unplanned, unreflective betting that undermines responsible gambling. Further research is urgently needed to understand their impact on betting behaviour, gambling harm, and consumer protection, with this study providing a foundation for future work and regulation. |

| Study | Authors | Year | Setting | DOI (for studies) URL (for reports) | Record type | MMAT | Study design | Advertising types examined / Marketing channels | Restriction/ policy examined | Restriction/ policy document(s) | Findings/outcomes | Restriction/policy implications | Policy recommendations | Knowledge gaps (future research) |
|---|---|---|---|---|---|---|---|---|---|---|---|---|---|---|
| | | | | | | | | | | DIT_Commercial-Radio-Code-of-Practice.pdf)<br>• TV (https://www.acma.gov.au/publications/2019-10/rules/commercial-television-industry-code-practice-2015) | | | | |
| Hörnle et al., 2019 | Hörnle, J., Schmidt-Kessen, M., Littler, A., & Padumadasa, E. | 2019 | UK | https://doi.org/10.1080/13600834.2019.1664001 | Commentary | N/A | N/A | Online Advertisement of Online gambling | Committee of Advertising Practice, Advertising Standards Authority, Data Protection Act 1998 and the Privacy and Electronic Communications Regulations 2003, EU data protection law. | • Cap Code (https://www.asa.org.uk/static/c6be0fb9-2c66-4248-ba5b824bf26fd3d3/e8d86fa9-9b16-42db-a6d6ecd93cf8cda0/The-CAP-Code.pdf)<br>• Data Protection Act 1998 (https://www.legislation.gov.uk/ukpga/1998/29/pdfs/ukpga_19980029_en.pdf)<br>• EU data protection law (https://www.legislation.gov.uk/uksi/2003/2426/pdfs/uksi_20032426_en.pdf) | The paper highlights that online gambling advertising, particularly on social media, differs from traditional advertising by using data-driven targeting, influencers, and embedded content, making it less visible but more persuasive. Key concerns include the targeting of vulnerable users, such as young people or people at risk of gambling harm, and the lack of transparency when marketing is disguised within user-generated or influencer content. Social media platforms rely on notice-and-takedown enforcement, placing responsibility on users rather than proactively policing advertising, which creates regulatory gaps. The sophisticated technology behind online advertising prioritises economic efficiency over ethics, consumer autonomy, or data protection, resulting in a complex ecosystem that current laws struggle to regulate effectively. | Current data protection frameworks rely heavily on user consent, but this offers limited protection. Consumers face information overload, and even when they have the option to withhold consent, the volume and complexity of data collection for marketing makes it impossible to effectively control how their data is used | Stronger, more comprehensive, and innovative regulations are needed to protect consumers in digital gambling environments. Consumer protection laws prohibiting undue influence, pressure selling and unfair commercial practices are more suited to address the harms. Social media platforms should require users to clearly disclose commercial relationships with gambling advertisers and prohibit undisclosed advertising. Additionally, policy reforms should implement safeguards beyond consent to protect users from exploitative targeted advertising. | Future studies should investigate how online advertising systems comply with data protection laws, focusing on data-sharing practices within complex digital advertising ecosystems. The authors note, the complexity of such research and the challenges it would face. |
| Jääskeläinen et al., 2025 | Jääskeläinen, P. P., Väkeväinen, E. V., & Marionneau, V. K. | 2025 | Finland | https://doi.org/10.1177/14034948251394913 | Empirical Study | ***** | Mixed-method online survey (N = 132), using cross-tabulation and bivariate correlations, and thematic analysis. | Exposure to Marketing | Finnish gambling legislation state monopoly system, New licensing system Finland (2027) | • Official Finnish legislation 2001 (https://www.finlex.fi/en/legislation/2001/1047?language=fin&highlightId=993687&highlightParams=%7B%22type%22%3A%22BASIC%22%2C%22search%22%3A%221047%2F2001%22%7D#chp_1__sec_1)<br>• New proposed framework (https://www.finlex.fi/en/government-proposals/2025/ | Bivariate correlations indicated significant associations between operator type and gender, age, and self-reported gambling problems. Men and younger participants were more likely to gamble with offshore providers or both Veikkaus and offshore operators, with offshore gamblers more likely to report past or current gambling problems. Despite marketing restrictions, exposure to gambling marketing was widespread, with most participants reporting seeing online marketing from both Veikkaus and offshore providers. Marketing from both sources was perceived as extensive and increasingly aggressive. Of the participants, 60.5% indicated wanting stricter marketing regulations or a blanket ban on gambling marketing. | This study has shown that despite recent regulatory restrictions on gambling marketing in Finland, individuals who gamble actively are exposed to widespread and increasingly aggressive gambling marketing by the monopoly provider and by offshore operators. The upcoming licensing system is likely to further increase the visibility of marketing. | Partial restrictions may push operators to intensify use of remaining marketing channels, suggesting that full prohibitions may be more effective. This approach was supported by respondents, favouring strict restrictions or a complete ban. Prioritising public health over commercial interests is key to reducing gambling-related harm. In Finland, marketing is expected to increase under the new licensing system and relaxed regulations, despite limited public preference for greater exposure. | Future research should examine how different types of gambling marketing influence harm and consumption, recognising that impacts may vary across population groups. Longitudinal studies are also needed to track how marketing changes as Finland shifts from a monopoly to a licensing model, helping to understand how policy decisions shape individual experiences and exposure |

| Study | Authors | Year | Setting | DOI (for studies) URL (for reports) | Record type | MMAT | Study design | Advertising types examined / Marketing channels | Restriction/ policy examined | Restriction/ policy document(s) | Findings/outcomes | Restriction/policy implications | Policy recommendations | Knowledge gaps (future research) |
|---|---|---|---|---|---|---|---|---|---|---|---|---|---|---|
| | | | | | | | | | | 16?language=swe&highlightId=977853&highlightParams=%7B%22type%22%3A%22BASIC%22%2C%22search%22%3A%22gambling%22%7D) | | | | |
| Johns et al., 2017 | Johns, R., Dale, N., Alam, S. L., & Keating, B. | 2017 | Australia | https://researchsystem.canberra.edu.au/ws/portalfiles/portal/33066225/Impact_of_gambling_warning_messages_on_advertising_perceptions.pdf | Empirical study/ Report | **** | Mixed methods: Study 1- Analysis of secondary sources; Study 2- content analysis of Australian television advertisements (N = 280); Study 3- cross sectional online survey (N = 964) | Generic Gambling advertisement and Warning messages | Gambling Act (2001), Gambling Act (1998), Interactive Gambling (Player Protection) Act of Queensland (1998), Gambling Regulation Act of Victoria (2003) | • Interactive Gambling Act 2001 (https://www.legislation.gov.au/C2004A00851/latest/downloads)<br>• Queensland Interactive Gambling (Player Protection) Act 1998 (https://www.legislation.qld.gov.au/view/pdf/inforce/current/act-1998-014)<br>• Gambling Regulation Act 2003 Victoria (https://content.legislation.vic.gov.au/sites/default/files/2025-10/03-114aa110-authorised.pdf) | Study 1 highlights that gambling regulation is complex and inconsistent. While protections exist for minors, vulnerable groups, and misleading advertising, public expectations are not fully met, indicating a need for tighter controls on misleading claims and further improvements to maintain Australia's regulatory leadership. Study 2 found that gambling advertisements predominantly used informational appeals (67%), targeted experienced gamblers (46%), featured non-immediate calls to action (80%), and frequently used incentive-based messaging. Study 3 identified two audience segments with different responses to gambling adverts. Segment 1 (mostly people experiencing significant gambling harms*) was most influenced by calls to action, followed by appeal type, situation, and goal orientation. Segment 2 (mostly people gambling at moderate risk) was most influenced by appeal type, then situation, goal orientation, and call to action. The study also showed that well-designed warning messages can significantly reduce the perceived impact of gambling advertising. | Australia has a fairly comprehensive regulatory framework compared with most other OECD countries. However, there is a clear mismatch between public expectations and existing legislation. Current gaps leave gambling advertising largely self-regulated. For example, guidance from regulators on the wording and format of warning messages varies across jurisdictions, with some legislation failing to specify what should be included in these statements. Additionally, findings in study 3 highlight a key focus for regulators, ensuring that people gambling at moderate risk (Segment 2) remain actively engaged in processing gambling-related information. | A national framework would greatly assist in regulating gambling advertising, with careful consideration of how legislation is enforced across different jurisdictions. Future legislation should tighten provisions on deceptive marketing and provide clearer, more consistent guidelines to reduce the normalisation of gambling, particularly among children. Consideration should also be given to following Ireland's lead and introducing a complete ban on gambling advertising. Regulators could use these findings to develop better-targeted and more effective counter-advertising campaigns, as well as regulatory interventions. | Future research could potentially target people experiencing significant gambling harms* to better understand the triggers that activate their active processing of warning messages |
| Kroon, 2021 | Kroon, Å. | 2021 | Sweden | https://doi.org/10.1080/14680777.2021.1916771 | Empirical | **** | Qualitative, case study of a contemporary video ad campaign, inductive approach. | Ad series from Svenska Spell | Swedish Gambling Act (from 2019); The Marketing Act. | • Swedish Gambling Act (2018) in force 2019 (https://www.spelinspektionen.se/globalassets/dokument/engelska/gambling-act-sfs-2018_1138-uppdate-sfs-2024_255.pdf) | The results indicate that women are explicitly targeted through both masculine and feminine semiotic strategies, but in a relatively "moderate" and normalised way. Male audiences are addressed using stereotypically masculine framings, though without overtly macho or aggressive codes. Overall, the messaging promotes ordinary, everyday lifestyles, presenting gambling as normalised and low-risk. Svenska Spel recognises gambling as a practice engaged | The analysis further exposes that current regulations only partly cover other potential problems in ad design, such as ethnic stereotyping. It is argued that the law's demand for moderation in advertising may backfire as a strategy to protect people from the harmful effects of gambling. According to the Swedish Marketing Practices Act, marketing shall not be misleading, | N/A | More research should qualitatively explore the subtle and complex meaning-making of gambling ads in relation to their regulatory frameworks in order to uncover the many nuances of normalising gambling in society. More research on gender in advertising in online contexts should be conducted |

| Study | Authors | Year | Setting | DOI (for studies) URL (for reports) | Record type | MMAT | Study design | Advertising types examined / Marketing channels | Restriction/ policy examined | Restriction/ policy document(s) | Findings/outcomes | Restriction/policy implications | Policy recommendations | Knowledge gaps (future research) |
|---|---|---|---|---|---|---|---|---|---|---|---|---|---|---|
| | | | | | | | | | | | in by both genders, despite it often being framed as masculine. The two distinct advertisements also target different female audiences, one aimed at middle-class, middle-aged Swedish women aligned with "the Swedish way of life," and another ("The Bus") directed at younger, more ethnically diverse women. The broader campaign narrative emphasises "giving back to society," positioning the company as a philanthropic actor contributing to social goods such as youth services, infrastructure, and education. | and the gambling industry's ethical guidelines state that marketing shall not claim or imply that it is risk-free to gamble. Whilst Svenska Spel does not overtly mislead or claim that gambling is without risk, it more than implies that to gamble is to do a thoroughly good deed that benefits society as well as enables more gambling addiction research. | | |
| Lamont et al., 2011 | Lamont, M., Hing, N., & Gainsbury, S. | 2011 | Across jurisdictions but mainly Australia | https://doi.org/10.1016/j.smr.2011.04.004 | Report | ***** | Conceptual framework for research and regulatory review | Sport Sponsorships | UK Gambling Act 2005, timeslots watched by youth ban (Watershed ban) and use of logos on merch for children. | • UK Gambling Act 2005 (https://www.legislation.gov.uk/ukpga/2005/19/pdfs/ukpga_20050019_en.pdf) • CAP Code regarding under 18 (https://www.asa.org.uk/static/d9dd9d06-00e7-4630-81d460b598c7d976/gambling-and-lotteries-advertising-protecting-under-18s.pdf) • UK License conditions and Code of practice (https://www.gamblingcommission.gov.uk/licensees-and-businesses/lccp/print) • Watershed Ban (https://bettingandgamingcouncil.com/uploads/22703-BGC-IGRG-Code-7th-Edition-111023.pdf) | Sponsorship enables commercial gambling providers (CGPs) to reach large audiences, including youth and other vulnerable groups, thereby normalising gambling and potentially increasing the risk of problem gambling. CGPs have capitalised on sport sponsorship as an effective mechanism to meet marketing objectives, and evidence indicates that greater exposure to gambling advertising raises the likelihood of youth participation. Empirical research on gambling promotion through sport sponsorship remains limited, constraining the development of informed public policy. The proposed stakeholder-based framework offers a roadmap for future research and regulation, highlighting four key stakeholders: governments and regulators, the sponsored party (the 'property'), the sponsoring company, and the general public. | While some regulations exist, such as limiting exposure to minors, regulatory frameworks remain inconsistent and often reactive. Recent policy changes indicate that lawmakers are beginning to consider the role of sport sponsorship, and further legislation could substantially impact the management of sport. | These findings highlight the need for public health measures that protect individuals, promote safer gambling environments and products, and reduce harmful exposure to gambling. | Future research and regulation should examine the impact of gambling sponsorship in sport on vulnerable populations, with clearer differentiation between activities based on harm potential. Other key areas include effects on gambling behaviour, sports organisations' finances, and audience perceptions, alongside how decision-makers balance ethical, economic, and legal considerations in sponsorship agreements. Research should also assess whether gambling sponsorship enhances or damages the sport's image, influences fan base size, or affects viewing audiences. In addition, research into mechanisms that could reduce exposure among vulnerable groups would strengthen public health approaches. Overall, the proposed framework highlights multiple avenues for future research development. |
| Lopez-Gonzalez & Griffiths, 2016 | Lopez-Gonzalez, H., & Griffiths, M. D. | 2016 | Europe | https://doi.org/10.1089/glre.2016.2064 | exploratory overview/ Report | N/A | Critical analysis of existing literature and policy | In-Play betting advertising | European Commission (live in-play betting has not been individually addressed within), UK Gambling Act | • EU Legislation Consolidated (https://eur-lex.europa.eu/legal-content/EN/TXT/PDF/?uri=CELEX:12012E/TXT) | In-play or live betting occurs during events and has shifted gambling from a discontinuous to a continuous activity, increasing problem gambling risk. It accounts for around 50% of UK betting and is growing through gamification and fantasy sports, potentially | Live in-play betting has not been specifically addressed in European legislation, and EC recommendations rarely mention sports betting. Assessing whether advertisements comply with the law is | Player protection, commercial communication, and advertising are central to regulation. | N/A |

| Study | Authors | Year | Setting | DOI (for studies) URL (for reports) | Record type | MMAT | Study design | Advertising types examined / Marketing channels | Restriction/ policy examined | Restriction/ policy document(s) | Findings/outcomes | Restriction/policy implications | Policy recommendations | Knowledge gaps (future research) |
|---|---|---|---|---|---|---|---|---|---|---|---|---|---|---|
| | | | | | | | | | 2005, Gambling (Licensing & Advertising) Act 2014, and UK broadcast/non-broadcast advertising codes, Spain draft legislation 2016, European Commission 2011 Green Paper, Recommendation on Principles for the Protection of Consumers and Players of Online Gambling Services and for the Prevention of Minors from Gambling Online. | • UK Gambling Act 2005 (https://www.legislation.gov.uk/ukpga/2005/19/pdfs/ukpga_20050019_en.pdf) • Gambling Licensing & advertising Act 2014 (https://www.legislation.gov.uk/ukpga/2014/17/pdfs/ukpga_20140017_en.pdf) • UK Cap Code (https://www.asa.org.uk/static/c6be0fb9-2c66-4248-ba5b824bf26fd3d3/e8d86fa9-9b16-42db-a6d6ecd93cf8cda0/The-CAP-Code.pdf) • UK BCAP code (https://www.asa.org.uk/static/846f25eb-f474-47c1-ab3ff571e3db5910/717b1f78-c8b3-4e5b-b49f337f16bd1cb0/BCAP-Code-full.pdf) • Spain Draft bill leading to Royal decree (https://www.boe.es/buscar/pdf/2020/BOE-A-2020-13495-consolidado.pdf) • EU commision Green paper 2011 (https://eur-lex.europa.eu/legal-content/EN/TXT/PDF/?uri=CELEX:52011DC0128) • EU Commision reccomendations (https://eur-lex.europa.eu/legal-content/EN/TXT/PDF/?uri=CELEX:32014H0478) | overtaking traditional forms. While public policy focuses on game integrity and protecting vulnerable groups, advertising content receives less attention. The European Commission's "Recommendation on Principles" and subsequent UK and Spanish regulations have addressed this, but empirical evidence on advertising effects is limited. Evidence on the impact of advertising is limited, though Australian studies suggest it can influence in-play betting behaviour. | challenging, as regulations are conservative and often lack scientific support. Promotions like free bets remain highly attractive, and regulation struggles to keep pace, leaving gamblers reliant on both regulatory oversight and operators' self-regulation. Certain regulations (e.g., banning integrated ads or celebrity endorsements) reflect research insights, but others (e.g., attractiveness effects, social influence) remain under-addressed. Both UK and Spanish regulations target different narratives; influence of attractive non-expert presenters (UK covers attractiveness; Spain does not), Celebrity endorsements (Spain prohibits; UK does not), Group/peer pressure dynamics, which can normalise gambling, are only partially addressed in both. | | |
| McGrane et | McGrane, | 2024 | UK | https:// | Empirical | **** | Quantitative | TV | Whistle-to- | • Whiste-to- | The whistle-to-whistle (W2W) | Voluntary partial | Partial restrictions like the | Future research on |

| Study | Authors | Year | Setting | DOI (for studies) URL (for reports) | Record type | MMAT | Study design | Advertising types examined / Marketing channels | Restriction/ policy examined | Restriction/ policy document(s) | Findings/outcomes | Restriction/policy implications | Policy recommendations | Knowledge gaps (future research) |
|---|---|---|---|---|---|---|---|---|---|---|---|---|---|---|
| al., 2024 | E., Pryce, R., Field, M., & Goyder, E. | | | doi.org/10.1080/16066359.2024.2444224 | | | secondary data analysis of TV scheduling and gambling advertising between September–December 2018 (pre-W2W ban) and 2019 (post-W2W ban), analysed using linear regression models | Advertising specifically during sports, Live and non-live | whistle ban | whistle ban (https://www.gov.uk/government/publications/high-stakes-gambling-reform-for-the-digital-age/high-stakes-gambling-reform-for-the-digital-age) | ban was associated with a decrease in gambling advertising around live football and other live sports, except for horse racing, where advertising increased. For non-live football programming, there was a marginal and statistically insignificant reduction in advertising between 2018 and 2019, while reductions around other non-live sports were small but statistically significant. Across other types of programming, advertising showed only minor changes: there were marginal reductions around documentaries, drama, entertainment, leisure, music, and other programming, whereas news and film programming experienced small increases in advertising. | gambling advertising restrictions were associated with a reduction in television advertising across all live sports, except horse racing where advertising increased. Despite the observed reductions in advertising during live sports, these programs (particularly live football) still carry some of the highest numbers of advertisements per program, and per-hour of programming, compared to the rest of the UK TV network. | W2W ban may be less effective than broader daytime advertising limits, findings highlight the potential value of stricter or more comprehensive policies to reduce gambling-related harm | trends in gambling advertising would be a valuable addition to the evidence base. Research should examine other forms of gambling advertising not covered by the W2W ban, such as online and embedded advertising, to assess whether reductions in TV advertising are offset by increases elsewhere. Additionally, research should investigate the relationship between exposure to gambling advertising around live sports and betting behaviour, as current data does not allow the assessment of potential dose-response effects. |
| McGrane et al., 2025 | McGrane, E., Pryce, R., Wilson, L., Field, M., & Goyder, E. | 2024 | UK | https://doi.org/10.1080/16066359.2024.2355183 | Empirical | **** | Quantitative secondary data analysis of changes in gambling advertising during live football games (N = 1,049) over three months (September–December) pre- (2018) and post-W2W (2019) ban, using inverse probability weighted (IPW) matching models | TV Advertising during Live football | Whistle-to-whistle ban | • Whiste-to-whistle ban (https://www.gov.uk/government/publications/high-stakes-gambling-reform-for-the-digital-age/high-stakes-gambling-reform-for-the-digital-age) | The implementation of the ban was associated with an overall reduction in advertising, driven primarily by decreases during half-time. However, It was associated with an increase in advertisements during the pre-match section. In the post-ban period, an average of three advertisements per programme remained. Although there were fewer advertisements on Sky and TNT Sports compared to ITV, these differences were not statistically significant. Notably, the industry did not substitute restricted-period advertisements with permitted lottery or bingo adverts. | The ban led to an overall reduction in TV gambling advertisements around live football games during the restricted periods, with a small increase in the unrestricted pre-match section and no change post-match. These findings suggest that TV advertising restrictions can be an effective policy tool for reducing the frequency of gambling adverts around live football, but partial bans may be less effective at reducing overall advertising prevalence on TV. | N/A | Future research should examine how the introduction and timing of gambling advertising restrictions affect overall exposure across different channels and programming, including live sports not subject to the ban, other TV genres, and pre- and post-game periods, as well as how these changes translate to gambling behaviour. Evidence on the subsequent behavioural impact of the ban is also required. |
| McKevitt et al., 2023 | McKevitt, S., White, M., Petticrew, M., Summerbell, C., Vasiljevic, M., Boyland, E., Cummins, S., Laverty, A. A., Millett, C., de Vocht, F., Junghans, C., & Vamos, E. P. | 2023 | UK ( England ) | https://doi.org/10.1093/pubmed/fdad155 | Empirical | **** | Quantitative census with local Authorities (LAs) (N = 333) exploring local commercial advertising policy, presence, commodity, frequency, type, and associations with LA characteristics. | Commercial Advertising and sponsorship | Advertising Standards Authority UK Code of Non-broadcast Advertising, Sales Promotion and Direct Marketing (CAP code), Gambling Act 2005 | • Cap Code (https://www.asa.org.uk/static/c6be0fb9-2c66-4248-ba5b824bf26fd3d3/e8d86fa9-9b16-42db-a6d6ecd93cf8cda0/The-CAP-Code.pdf) • UK Gambling Act 2005 (https://www.legislation.gov.uk/ukpga/2005/19/pdfs/ukpga_20050019_en.pdf) | Only a third (106) of LAs in England had a relevant policy (32%). These included restrictions on tobacco (91%), gambling (79%), alcohol (74%) and/or less healthy foods (24%).The most common harmful commodity considered was tobacco (91%), followed by gambling (79%). Policy prevalence was lowest in the East of England (22%), North East (25%) and North West (27%), higher in urban areas (36%) than rural areas (28%) and lower in the least (27%) compared with the most (38%) deprived areas. Definitions in policies varied, terminology and | English LAs currently underutilise their levers to reduce the negative impacts of harmful commodity industry marketing. Advertisement and sponsorship constraints were often influenced by and referred to national-level regulations or guidance. | This research provides examples for LAs that are considering implementing policies. Developing a consistent, unified approach across local authorities could support wider adoption of effective population-level policies addressing the commercial determinants of health. In the meantime, standardised guidance, featuring clear definitions, practical examples, and training resources, is needed to support consistent and effective implementation | This research provides examples for LAs that are considering implementing policies and a baseline for future research and evaluations. Future research should evaluate the impact, content, and implementation of policies across local authority contexts, particularly in relation to deprivation. Given the greater impact of Harmful Commodity Industry in deprived areas, it is important to |

| Study | Authors | Year | Setting | DOI (for studies) URL (for reports) | Record type | MMAT | Study design | Advertising types examined / Marketing channels | Restriction/ policy examined | Restriction/ policy document(s) | Findings/outcomes | Restriction/policy implications | Policy recommendations | Knowledge gaps (future research) |
|---|---|---|---|---|---|---|---|---|---|---|---|---|---|---|
| | | | | | | | | | | | scope with regard to gambling was also largely, with some explicit clauses for exceptions, such as the National and local society/authority lotteries | | across England. | assess whether policy approaches are proportionate to population need, while also identifying key facilitators and barriers to adoption, including stakeholder perspectives and industry influence. |
| Nairn & Rossi, 2021 | Nairn, A., & Rossi, R. | 2021 | UK | https://doi.org/10.1089/glr2.2021.0015 | Empirical | ***** | mixed-method content analysis | Social media gambling advertising | Committee of Advertising Practice (CAP) code, change the wording of CAP Code 16.3.12, Industry Group for Responsible Gambling (IGRG) Voluntary Code & Committee of Advertising Practice ((B)CAP) & Gambling Act 2005 | • Cap Code 16.3.12 (https://www.asa.org.uk/static/c6be0fb9-2c66-4248-ba5b824bf26fd3d3/e8d86fa9-9b16-42db-a6d6ecd93cf8cda0/The-CAP-Code.pdf Page 93)<br>• IGRG voluntary code (https://www.gamblingcommission.gov.uk/licensees-and-businesses/print/advertising-marketing-rules-and-regulations)<br>• UK Gambling Act 2005 (https://www.legislation.gov.uk/ukpga/2005/19/pdfs/ukpga_20050019_en.pdf) | Overall, regulatory compliance for organic Twitter advertising was very poor, with 74% of esports betting adverts and 68% of other gambling ads failing to meet at least one of the assessed benchmarks. Additionally, the study identified eight key regulatory failures in esports gambling advertising: the use of under-25s in adverts, the posting of late-night advertisements, the promotion of immediate betting opportunities, the absence of social responsibility messaging, the failure to clearly label content as advertising, the widespread use of unregulated content marketing, vague and ineffective rules regarding appeal to children, and high levels of engagement from children and young people. | There are substantial regulatory shortcomings for esports gambling advertising. The regulations are not straightforward, and they are not all in one place. Additionally, there are no specific social media regulations in the CAP Code. | Social media advertising requires a fundamentally new form of regulation, the authors call for increased attention to esports, the development of social media–specific regulations, improved transparency and responsibility in advertising, and the inclusion of young people in shaping regulatory frameworks. The UK government review of the Gambling Act is a perfect opportunity to create new, social media–specific regulations. | Enquiries strongly suggests that those drafting current legislation have scant knowledge of the esports industry ecosystem and the role of gambling within it. Additionally, a more objective measure of whether social media gambling advertising appeals to children is whether they follow gambling accounts and share or like the content. To the researcher's knowledge the adult CAP code creators and enforcers have never asked children or young people what is and is not appealing to them. |
| Newall et al., 2019 | Newall, P., Thobhani, A., Walasek, L., & Meyer, C. | 2019 | UK | https://doi.org/10.1371/journal.pone.0216876 | Empirical | ***** | Quantitative, Content Analysis of 2018 World Cup's matches ( N = 64) | Live-odds Advertising | UK gambling advertising regulations. | • Regulatory statement gambling advertising guidance (https://www.asa.org.uk/static/uploaded/5e19ecb5-a0b2-4322-aeb13047bb408298.pdf) & (https://www.gov.uk/government/publications/high-stakes-gambling-reform-for-the-digital-age/high-stakes-gambling-reform-for-the-digital-age) | During the 2018 FIFA World Cup, 69 live-odds gambling adverts were broadcast across 32 ITV matches, averaging just over two adverts per game and coming from five bookmakers. 39.1% of adverts could be resolved before the match ended, 24.6% featured improved odds, and 15.9% subset included "flash odds, creating a sense of urgency for viewers to bet quickly. Most adverts promoted complex betting markets, such as exact scores or player-specific outcomes, rather than simple win/draw/lose options, making them harder for consumers to evaluate. Overall, the study highlights that live-odds advertising is frequent, time-pressured, and focused on complex bets, raising significant consumer protection concerns despite existing UK regulatory | The research identified two recurring features which seem particularly relevant to recent regulatory guidance on "impulsiveness and urgency". | In comparison to calorie or alcohol unit labelling, to include numerical information that compares the risk of different football bets, which may help consumers understand complex probabilities. | To determine how targeted content might impact the behaviours of gamblers, particularly in the UK. This study was limited to TV gambling advertising and the data for online advertising is held by gambling operators and media platforms, which would be useful to investigate if data were made available. |

| Study | Authors | Year | Setting | DOI (for studies) URL (for reports) | Record type | MMAT | Study design | Advertising types examined / Marketing channels | Restriction/ policy examined | Restriction/ policy document(s) | Findings/outcomes | Restriction/policy implications | Policy recommendations | Knowledge gaps (future research) |
|---|---|---|---|---|---|---|---|---|---|---|---|---|---|---|
| | | | | | | | | | | | guidance. | | | |
| Newall & Xiao, 2021 | Newall, P. W., & Xiao, L. Y. | 2021 | UK, Spain, Italy | https://doi.org/10.1089/glr2.2021.0027 | Commentary | N/A | N/A | Marketing bans in professional sports and emergence of trading or cryptocurrency | Full prohibition in Italy (e.g., Dignity Decree 2019); prohibition of sports team sponsorship and advertising between 1am and 5am in Spain (Royal Decree 2018); and the UK's consideration of banning sports sponsorships. | • Italy Dignity Decree (https://www.normattiva.it/uri-res/N2Ls?urn:nir:stato:decreto.legge:2018-07-12;87) • Spain Royal Decree (https://www.boe.es/buscar/pdf/2020/BOE-A-2020-13495-consolidado.pdf) | The authors argue that there is a trend towards sports sponsorship of gambling-like industries such as trading apps and cryptocurrencies, which are not affected by the bans. Following the ban in Spain, all gambling sponsorships of La Liga football teams were removed, which six out of 20 previously had, with two clubs having trading app sponsorships on shirts. In Italy, some football teams were already sponsored by cryptocurrencies and financial trading apps, highlighting the new trend towards these industries for sponsorship. Two football clubs in the men's English Premier League were sponsored by cryptocurrency gambling operators, which did not have a license to operate in the UK | Restrictions on gambling sponsorships may open the way for gambling-like industries, such as trading apps and cryptocurrencies. | Restrictions should be placed on gambling-like industries such as trading apps and cryptocurrencies as they help to legitimise the use of these products, making them appear less risky than gambling. | To investigate emerging industries to allow policymakers to make informed decisions. |
| Nyemcsok et al., 2018 | Nyemcsok, C., Thomas, S. L., Bestman, A., Pitt, H., Daube, M., & Cassidy, R. | 2018 | Australia | https://doi.org/10.1556/2006.7.2018.128 | Empirical Study | **** | Mixed-methods, interviewer-assisted surveys including young people aged 11-16 years of age (N = 111). Same sample and data as Thomas et al. (2018). | Gambling advertising to young people | Australian Federal Government prohibition on gambling advertising during live sports from 5 minutes before until 5 minutes after, up until 8.30pm. Regulation on gambling advertising between 6am and 8.30am, and 4pm and 7pm outside of live sports. | • Enacted from 2027 (https://www.pm.gov.au/media/strong-action-tackle-gambling-harms) | The majority of young people recalled at least one gambling brand (n = 84.6%) and over three quarters of young people recalled at least one sports betting brand (81.1%). Most of the young people also associated the primary brand colouring with at least one brand (91.9%). Young people were able to recall promotions that they had seen, while some were aware of the strategies that were used. Young people also reported that inducement promotions were the most influential. A fifth (22.5%) reported that they would bet when they turned 18, with boys being more likely compared to girls (p = .001). | The current regulations in Australia are ineffective in preventing young people's recall and awareness of gambling brands, indicating that further measures are needed. | To limit gambling promotions across all media platforms and limit the depictions of inducements that create perceptions that risks from gambling can be reduced | Whether young people's attitudes towards gambling changes as they turn to the legal age of gambling and what factors influence that change. |
| Pitt et al., 2024 | Pitt, H., McCarthy, S., Randle, M., Arnot, G., Daube, M., & Thomas, S. | 2024 | Australia | https://doi.org/10.1186/s12889-024-19331-x | Empirical Study | **** | Qualitative, focus groups (n = 22) with 12-17 year olds (n = 64). | Gambling marketing to young people | Timing and placement restrictions in Australia, including a ban on advertising near schools and on public transport, and during children's television viewing hours. | • Enacted from 2027 (https://www.pm.gov.au/media/strong-action-tackle-gambling-harms) | Young people wanted more effective regulation addressing the content and frequency of gambling marketing. Some noted that current restrictions, where gambling marketing was allowed after 7pm, were not effective as they stay up later than 7pm. Participants also discussed the importance of age verification on social media platforms including YouTube, where those below a certain age would not be shown gambling marketing. However, others mentioned still seeing gambling marketing even though they had entered an age of under 18. Other participants called for outright bans on gambling | The current timing restrictions were felt to be ineffective by participants. While current regulations restrict timing and placement, young people expressed concerns related to the volume of gambling marketing across all forms of media. Current regulations were deemed outdated, particularly when related to social media. | To implement tighter restrictions overall such as outright bans or specific regulations for social media such as better age gating. | To ensure that girls are invited into studies related to gambling and gambling marketing and to explore social factors such as parental gambling attitudes or behaviours. |

| Study | Authors | Year | Setting | DOI (for studies) URL (for reports) | Record type | MMAT | Study design | Advertising types examined / Marketing channels | Restriction/ policy examined | Restriction/ policy document(s) | Findings/outcomes | Restriction/policy implications | Policy recommendations | Knowledge gaps (future research) |
|---|---|---|---|---|---|---|---|---|---|---|---|---|---|---|
| | | | | | | | | | | | marketing. | | | |
| Planzer et al., 2014 | Planzer, S., Gray, H. M., & Shaffer, H. J. | 2014 | Europe | https://doi.org/10.1016/j.ijlp.2013.11.002 | Empirical Study | **** | Quantitative, a presentation of policies from European countries (N = 30) and a analysis of prevalence rates from some of those European countries (N = 12). | Gambling advertising generally | The restrictions on gambling advertising across EU member states | N/A | For all land-based games, many set no restrictions through the study period (43-57%) and jurisdictions rarely had a consistent state of prohibition (7-23%). Some jurisdictions (17-20%) experienced changing regulations over time. For online games, few set no restrictions (27-37%) compared to land-based games. Jurisdictions were more likely to experience a consistent state of prohibition (27-43%). The majority (82%) of jurisdictions did not change their advertising policies over time. Overall, advertising restrictions were rare. | More restrictions (not bans) on the advertising of online games were associated with lower rates of disordered gambling. The authors questions whether the gambling policies had any meaningful impact on the prevalence of gambling disorders. | European gambling policy making should be based on facts rather than assumptions. Each government should fund a credible body to document the costs of gambling. | To examine the extent in which environmental aspects impact gambling behaviour. To examine the relationship between policy and additional socio-economic indicators of gambling-related harms. |
| Purves et al., 2020 | Purves, R. I., Critchlow, N., Morgan, A., Stead, M., & Dobbie, F. | 2020 | UK | https://doi.org/10.1016/j.puhe.2020.02.012 | Empirical Study | ***** | Quantitative, frequency analysis of verbal and visual gambling marketing references during television broadcasts of football (n = 5), tennis, Formula 1, boxing and rugby union (each n = 1) from 2018. | Gambling marketing references during television broadcasts | Gambling Act 2005 and the voluntary whistle-to-whistle ban in televised sports | • Gambling Act 2005 (https://www.legislation.gov.uk/ukpga/2005/19/contents)<br>• White paper voluntary bans (https://www.gov.uk/government/publications/high-stakes-gambling-reform-for-the-digital-age/high-stakes-gambling-reform-for-the-digital-age) | There were 2,595 gambling marketing references across five football matches, an average of 519 per broadcast and 2.75 times per broadcast minute. Most (77%) were featured in-play and the most popular location was border-of-play (38%) such as pitch-side adverts. The most popular format was branded merchandise (41%). Gambling brand logos appeared in 99% of the references. Only 2% of references were advertisements during commercial breaks. Some advertisements were during half-time, which would not be permitted under the proposed whistle-to-whistle ban. There were references observed across boxing, rugby union, and tennis, but none in Formula 1. | The proposed voluntary ban would have little effect on the volume of gambling advertising in sports marketing. The authors argued that sponsorships would continue regardless of the voluntary ban because the locations include pitch-side hoardings and front of shirt. | Statutory and mandatory restrictions may be required to reduce exposure to gambling advertising, and provide greater protection to young and vulnerable groups in the UK. The authors also recommended a front-of-shirt ban or restricting advertising during a sporting event. | To include broadcasts of Scottish women's football in comparison to the men's game. |
| Regan et al., 2022 | Regan, M., Smolar, M., Burton, R., Clarke, Z., Sharpe, C., Henn, C., & Marsden, J. | 2022 | UK | https://doi.org/10.1016/S2468-2667(22)00137-2 | Empirical Study | **** | A three-round Delphi study and an implementation rating exercise with experts (N = 35). | Marketing and advertising across television, radio, social media, streaming, and sports sponsorships. | Gambling Act 2005 | • Gambling Act 2005 (https://www.legislation.gov.uk/ukpga/2005/19/contents) | A consensus was reached on 14 of the 15 measures (93%) related to marketing advertising, promotion, and sponsorship. Among these measures, six of the 15 received scores above 50% (judged as highly effective). The proposed bans were: Ban all price and discount promotions on gambling products and services, ban bet-to-view commerical arrangements, ban strategies that incentivise gambling or create a sense of urgency to bet, ban gambling advertising during television programmes aimed at children and during age-rated films, universal bans on all gambling marketing, advertising, and promotions, ban advertising and marketing on radio, television, social | As these were proposed restrictions, there were no implications. However, the study noted that changes to advertising and marketing were currently in the scope of the review of the Gambling Act 2005. | To follow a public health approach with a: ban on all price and discount promotions on gambling products and services, ban bet-to-view commerical arrangements, ban strategies that incentivise gambling or create a sense of urgency to bet, ban gambling advertising during television programmes aimed at children and during age-rated films, universal bans on all gambling marketing, advertising, and promotions, ban advertising and marketing on radio, television, social media, and streaming. | To involve experts on the basis of their knowledge and experience of gambling. |

| Study | Authors | Year | Setting | DOI (for studies) URL (for reports) | Record type | MMAT | Study design | Advertising types examined / Marketing channels | Restriction/ policy examined | Restriction/ policy document(s) | Findings/outcomes | Restriction/policy implications | Policy recommendations | Knowledge gaps (future research) |
|---|---|---|---|---|---|---|---|---|---|---|---|---|---|---|
| | | | | | | | | | | | media, and streaming. | | | |
| Regulus Partners, 2018 | Regulus Partners. | 2018 | Europe | https://www.eerstekamer.nl/overig/20191115/gambling_and_advertising_an/document | Technical Report | N/A | N/A | All forms of gambling advertising other than bonuses. | Sweden (Gambling Act 2018), Denmark (Act on Gambling 2010), Norway (Lottery Act, Gaming Schemes Act replaced by Gambling Act), Belgium (Gaming Act 1999 and The Royal Decree on Games of Chance, 2018), Italy (didn't take full effect until 2019 but is now the Dignity Decree 2019), Spain (a draft of the Royal Decree 2018), UK (Gambling Act 2005, CAP codes and ASA). | • Sweden Gambling Act 2018 (https://www.spelinspektionen.se/globalassets/dokument/engelska/gambling-act-sfs-2018_1138-uppdate-sfs-2024_255.pdf)<br>• Denmark (https://www.spillemyndigheden.dk/uploads/2019-01/Act%20on%20Gambling.pdf)<br>• Norway (Lottery Act: https://app.uio.no/ub/ujur/oversatte-lover/data/lov-19950224-011-eng.pdf)<br>• Gambling Act (https://lovdata.no/dokument/LTI/lov/2022-03-18-12)<br>• Belgium 1999 (https://www.gamingcommission.be/fr/legislation/loi-sur-les-jeux-de-hasard-du-7-mai-1999)<br>• Belgium 2023 (https://www.ejustice.just.fgov.be/cgi_loi/article.pl?language=fr&dt=KONINKLIJK+BESLUIT&nl=n&text1=kansspel&choix1=en&trier=afkondiging&lg_txt=f&type=&sort=&numac_search=2023030509&cn_search=&caller=list&&view_numac=2023030509dx2023030509fx2023030509nx2024021809nl)<br>• Italy Dignity Decree (https://www.normattiva.it/uri-res/N2Ls? | The report highlights variation in national regulatory frameworks. Sweden applies specific restrictions (e.g. age and bonus rules) but is described as subjective and lacking clarity. Denmark operates a liberal, principles-based system focused on fairness and preventing misleading or child-targeted advertising, with no limits on advertising volume. Norway has reduced mass-market gambling advertising in media, although access to licensed operators remains easy. Belgium moved from limited regulation under the 1999 Gaming Act to stricter controls in 2018, becoming an early adopter of tighter advertising rules. Italy introduced early online gambling regulation and later implemented a near-total advertising ban from 2019. Spain has a mixed system with national and regional governance, including proposed measures such as a 10pm–6am watershed and regional bans. The UK maintains a largely liberal, principles-based approach with relatively few restrictions, mainly watershed rules, with exceptions such as live sports and bingo. | For Spain, Demark, Belgium, Italy, it was unclear or not possible to assess whether restrictions had any impact at the time. However, it did state that Italy's partial ban did not slow down market growth. For Norway, while there were restraints on the volume and nature of advertising it stated that advertising was still likely to grow. For the UK, while changes were announced in 2018/19, they had not been implemented to have a noticeable impact on advertising. | Restrict the permission to advertise to domestically licensed operators. Gambling advertising should not target below the legal age to gamble, or those who are self-excluding, and it should not glamorise gambling. Steps should be taken to protect children from gambling advertising, which includes removing sponsorships on youth-size replica football kits. Athletes below the legal age to gamble should also not be allowed to represent gambling and endorsements should be banned in youth sports. | N/A |

| Study | Authors | Year | Setting | DOI (for studies) URL (for reports) | Record type | MMAT | Study design | Advertising types examined / Marketing channels | Restriction/ policy examined | Restriction/ policy document(s) | Findings/outcomes | Restriction/policy implications | Policy recommendations | Knowledge gaps (future research) |
|---|---|---|---|---|---|---|---|---|---|---|---|---|---|---|
| | | | | | | | | | | urn:nir:stato:decreto.legge:2018-07-12;87)<br>• Spain Royal Decree (https://www.boe.es/buscar/pdf/2020/BOE-A-2020-13495-consolidado.pdf)<br>• Gambling Act 2005 (https://www.legislation.gov.uk/ukpga/2005/19/contents) | | | | |
| Riley & Moimoi, 2025 | Riley, D., & Moimoi, E. | 2025 | UK | https://www.gambleaware.org/media/0xrns0z4/online-gambling-marketing-policy-paper.pdf | Policy report | N/A | N/A | Gambling marketing and online content | Gambling Act 2005, ASA, CAP codes | • Gambling Act 2005 (https://www.legislation.gov.uk/ukpga/2005/19/contents)<br>• CAP (https://www.asa.org.uk/static/b324a7dd-94d6-4fb2-979365d66acb2e36/7cc86b92-3ecc-4f1c-937b1cf2c85654d6/The-CAP-Code-Gambling.pdf) | The report highlights that children and young people are regularly exposed to online gambling marketing. They reference a statistic which showed that over three in five 11-17 year olds had seen or heard gambling advertising online platforms. The authors also argue that platforms heighten this exposure by their design (algorithms, marketing etc.). The report also highlights that online gambling marketing normalises gambling as harmless fun. Gambling marketing may also have an effect on those already experiencing gambling harm. | The regulatory system in the UK is inadequate to deal with gambling marketing in the digital age, as it primary focusses on self-regulation. There needs to be stronger accountability and communication between authorities such as the ASA, DCMS and DSIT. | Align and strengthen online safety powers and regulatory programmes, which includes ensuring a single entity has oversight and increase collaboration with the ASA. Increasing action from online platforms which includes increased accountability and age protections. Ensure existing programmes work towards reducing gambling harms, tighten self-regulation (such as tightening what "appeals" to children), and establish cross-government taskforces to explore policies. | N/A |
| Rossi et al., 2021 | Rossi, R., Nairn, A., Smith, J., & Inskip, C. | 2021 | UK | https://doi.org/10.1177/07439156219967 4 | Empirical Study (three studies) | ***** | Quantitative, big data analysis and a content analysis of gambling advertisements on Twitter | Gambling advertising, including content marketing, on social media (Twitter) | Evaluates all CAP codes which relate to gambling. CAP code 16. | • CAP Codes (https://www.asa.org.uk/static/b324a7dd-94d6-4fb2-979365d66acb2e36/7cc86b92-3ecc-4f1c-937b1cf2c85654d6/The-CAP-Code-Gambling.pdf) | Bookmakers were highly active on social media, averaging 14 tweets per day each, with one operator posting around 122 daily. A substantial proportion of traditional gambling ads (34%) strongly emphasised monetary rewards, potentially conflicting with CAP Code 16.3.4. Content appealing to younger audiences was also identified, 17% of e-sports ads and 3% of traditional gambling ads were considered particularly attractive to children (CAP Code 16.3.12), while 76% of e-sports tweets used cartoons or animation contrary to updated guidance. Some traditional ads also featured individuals appearing under 25, including two well-known e-sports players, potentially breaching CAP Code 16.3.14. Transparency issues were evident, with 422 tweets lacking clear advertising labelling (CAP Code 2.1). Audience data further indicated that 73% of | In 42% of the cases, it was not clear whether e-sports advertising content was of particular appeal or not as the code is illogical.68% of traditional and 74% of e-sports advertisements contravened regulations, indicating a serious problem. | 1) address E-sports gambling advertising, 2) change of particular appeal code (16.3.12), 3) Use technology to exclude those under 18, 4) Better labelling of organic gambling advertisements and more social responsibility messages, 5) New social media-specific regulation, 6) Better enforcement of current regulations. | Create fake social media accounts whose feeds could be analysed to investigate demographic differences. Investigate the effects of content marketing on social media, especially focussing on the normalisation of gambling and its image. |

| Study | Authors | Year | Setting | DOI (for studies) URL (for reports) | Record type | MMAT | Study design | Advertising types examined / Marketing channels | Restriction/ policy examined | Restriction/ policy document(s) | Findings/outcomes | Restriction/policy implications | Policy recommendations | Knowledge gaps (future research) |
|---|---|---|---|---|---|---|---|---|---|---|---|---|---|---|
| | | | | | | | | | | | followers were under 24, with 68% of engagement from users under 24 and 28% of e-sports engagement coming from under-16s, raising questions about exposure and compliance in practice. | | | |
| Rossi et al., 2023 | Rossi, R., Wheaton, J., Moxey, M., & Tozzi, E. | 2023 | UK | https://www.bristol.ac.uk/media-library/sites/business-school/documents/BRISTOL-UNI-GAMBLING-Report2023-2.pdf | Technical Report | N/A | Quantitative, frequency analysis of English Premier League Games (N = 6), sports news and radio. A content analysis of gambling advertising on social media. | Gambling marketing on sports-related media. | Whistle-to-whistle and front of shirt bans, and restrictions on social media. | • Premier legue commitment (https://www.premierleague.com/en/news/3147426)<br>• Mentioned in White Paper (https://www.gov.uk/government/publications/high-stakes-gambling-reform-for-the-digital-age/high-stakes-gambling-reform-for-the-digital-age) | Across 10,999 recorded messages, gambling content was predominantly present during live match coverage (67.2%), with relatively little appearing during commercial breaks (3.45%). The most common exposure point was pitch side advertising (55.8%), followed by repeated branding before and after matches (10.68%) and on players' shirts (9.07%). Harm reduction and age restriction messaging were comparatively limited (14.5% and 11.1% respectively). Additional exposure occurred across other media: Sky Sports News featured 2,014 gambling messages, largely during match highlights, radio included 117 gambling messages, mainly during ad breaks (75.9%) and social media contained 1,902 gambling advertisements, including 1,129 paid ads and 773 organic posts on X, many of which functioned as content marketing. A notable proportion of conventional ads (around 35%) did not display age restrictions, and some social media posts were not clearly identifiable as advertising. | Self-regulation is ineffective and the front-of-shirt voluntary ban does not go far enough in reducing gambling messages and exposure. The whistle-to-whistle ban also has a limited effect due to identical references posted elsewhere, such as pre and post match. | Comprehensively regulate gambling messages in all forms during football matches, encompassing radio and social media. This would also need to include all visible areas, not just front of shirts. Strengthen social media regulations to ensure that content marketing is clearly labelled or banned. | N/A |
| Rossi & Nairn, 2024 | Rossi, R., & Nairn, A. | 2024 | UK | https://doi.org/10.1086/729290 | Empirical Study | ***** | Quantitative, an online survey with children aged 11-17 (n = 210), young people 18-24 years old (n = 222) and adults aged 25-78 (n = 221), which comprised of a self-assessment manikin (SAM) scale to measure emotional responses after viewing gambling advertisements on Twitter | Gambling advertising, including E-sports and content marketing, on social media (X) | Gambling Act 2005, and CAP code 16.3.12, should not be of strong appeal to children or young persons | • Gambling Act 2005 (https://www.legislation.gov.uk/ukpga/2005/19/contents)<br>• CAP (https://www.asa.org.uk/static/b324a7dd-94d6-4fb2-979365d66acb2e36/7cc86b92-3ecc-4f1c-937b1cf2c85654d6/The-CAP-Code-Gambling.pdf) | In the study, 45.2% of 11-17 year olds and 72.4% of 18-24 year olds reported seeing gambling advertising at least once a week on their social media feeds. Further, 19 out of 24 gambling advertisements were more appealing to children and young people than adults. In terms of emotional responses, 15 out of the 24 advertisements triggered positive emotions such as 'happiness' and 'delight'. Adults were four times more like than children and young people to have negative emotions such as 'distressed' and 'angry' when exposed to advertisements. Overall, when exposed to gambling advertisements, children and young people have positive emotions and adults have negative. | The authors argued that the current strong appeal codes apply to under 18, but the 18-24 year olds in this study showed the highest level of appeal towards gambling advertisements. They also argue that adults regulators are poor judges of is what of strong appeal. | To: include children in discussions when creating new guidance or code and consider their answers; expand 'young people' up until the age of 24; and ban all content marketing. | Take advertisements banned and ask children to assess them, replicate this study across countries, using other media (e.g., TikTok), and with other marketing strategies (influencers), use biometrics to measure appeal in children, young people, and adults such as galvanic skin response and heart rate, set up an experiment to assess whether content and Esports marketing prime children to gamble, and develop longitudinal studies to explore legal gambling. |
| Rossi et al., 2024 | Rossi, R., Wheaton, J., | 2024 | UK | https://www.bristol.a | Technical | N/A | Quantitative, frequency | Gambling marketing on | Voluntary Code of | • White paper referring to | This was a comparison to the 2023 report above and showed | Self-regulation is ineffective and the front- | A comprehensive whistle-to-whistle ban covering | N/A |

| Study | Authors | Year | Setting | DOI (for studies) URL (for reports) | Record type | MMAT | Study design | Advertising types examined / Marketing channels | Restriction/ policy examined | Restriction/ policy document(s) | Findings/outcomes | Restriction/policy implications | Policy recommendations | Knowledge gaps (future research) |
|---|---|---|---|---|---|---|---|---|---|---|---|---|---|---|
| | Moxey, M., Moradipour, S., Tozzi, E., & Singer, J. | | | c.uk/media-library/sites/business-school/documents/Premier_League_Report_2024.pdf | Report | | analysis of English Premier League Games (N = 6), sports news and radio. A content analysis of gambling advertising on social media. | sports-related media. | Conduct, including the whistle-to-whistle and front of shirt bans, and restrictions on social media. | policies (https://www.gov.uk/government/publications/high-stakes-gambling-reform-for-the-digital-age/high-stakes-gambling-reform-for-the-digital-age) | that advertisements had increased to 29,145 messages across the matches, news, radio, and social media. During the televised matches, there were 23,690 messages observed, with 39.5% occurring during the whistle-to-whistle ban. Gambling logos on front of players' shirts accounted for 8.3% of messages and sleeves 0.5%. Only 18.4% contained responsible messaging. There were 4,332 messages on Sky Sports News and 214 on the radio. On social media, there were 909 gambling advertisements posted. Of the organic advertisements (n = 632), 305 were content marketing. Content marketing included many visual references to athletes. 13% of content marketing and 2% of conventional advertisements were likely to have a strong appeal to children, breaching CAP code 16.3.12. There were many instances of content marketing not being obviously identifiable and thus also in breach. | of-shirt voluntary ban does not go far enough in reducing gambling messages and exposure. The whistle-to-whistle ban also has a limited effect. The new Code of Conduct has failed to address gambling marketing. | all forms of gambling marketing and sponsorships at sporting events. Looking towards jurisdictions such as the Netherlands, allowing clubs to find other sponsorships. Mandate that all gambling marketing should include responsible messaging that focuses on the potential harms of gambling (e.g., chances are). A full ban on content marketing. | |
| Rossi et al., 2024 | Rossi, R., Wheaton, J., Nairn, A., Moradipour, S., & Tozzi, E. | 2024 | USA | https://www.bristol.ac.uk/media-library/sites/business-school/documents/Bristol_SM_Research_Report.pdf | Technical Report | N/A | Quantitative, content analysis of gambling advertisements posted by four major gambling brands | Gambling advertising on social media platforms | Section 6, of the AGA Code "monitoring code compliance". Restrictions examined: below 21 years of age; inclusion of cartoon characters, or celebrities, influencers to music appealing to those under 21; inclusion of responsible messaging and a helpline; FCT act which prohibits unfair or deceptive acts. | • AGA code (https://www.americangaming.org/wp-content/uploads/2020/09/AGA-Responsible-SB-Marketing-Code.pdf) | A total of 1,663 gambling advertisements. Of which, 1,353 (81%) were organic advertising (53% content marketing; 48% conventional ads) and 310 (19%) were paid-for advertising (0 content marketing, 100% conventional). Content marketing advertisements were more successful in generating impression and likes than conventional. Two ads that accrued over 1 million impressions included fictional characters played by celebrities, and athletes. Only 337 (25%) included responsible gaming messages, 345 (26%) included help lines, and 341 (25%) included age restrictions. 75% of the 1,353 organic advertisements did not included one or more of the responsible messages, helplines, or age restrictions. Only 42% of organic advertisements were clearly identified as advertising. For paid-for advertisements, all restrictions were adhered to. | Poor compliance to the advertising regulations in the US, as only around 25% complied. These findings highlight the weaknesses in the current voluntary codes and show how companies do not adere to them. | 1) Federal legislation governing gambling marketing; 2) ban gambling content marketing on social media, 3) Strengthen AGA codes with detailed guidelines, 4) Include children in the gambling marketing policy-making, 5) Restrict the use of celebrities, 6) Establish a centralised database for online gambling ads | N/A |
| Rossi & Nairn, 2025 | Rossi, R., & Nairn, A. | 2025 | UK | https://doi.org/10.1177/147078532412929 53 | Empirical Study | ***** | Quantitative, online experiment with participants aged 11-78 | Gambling advertising on social media including content | CAP code 2.1, marketing must be obviously identifiable. | • Cap Code (https://www.asa.org.uk/static/c6be0fb9-2c66-4248-ba5b824bf26fd3 | Children and young adults show significantly lower recognition rates for social media gambling adverts than older adults. Irrespective of age, content marketing is challenging to | The study showed that content marketing is not obviously identifiable and therefore in breach of the CAP code 2.1. The regulations also lack a test | To ban all gambling content marketing, or an alternative which would include a message to stipulate that 'this is an advertisement'. To expand | To explore more ecologically valid methodologies such as real-time social media use, expand participant demographics to |

| Study | Authors | Year | Setting | DOI (for studies) URL (for reports) | Record type | MMAT | Study design | Advertising types examined / Marketing channels | Restriction/ policy examined | Restriction/ policy document(s) | Findings/outcomes | Restriction/policy implications | Policy recommendations | Knowledge gaps (future research) |
|---|---|---|---|---|---|---|---|---|---|---|---|---|---|---|
| | | | | | | | (N = 650) | marketing | | d3/e8d86fa9-9b16-42db-a6d6ecd93cf8cda0/The-CAP-Code.pdf) | identify compared to conventional adverts. Recognition levels for gambling content marketing are around chance for children and young adults. | for what is identifiable and is reliant on argument rather than data. | gambling advertising literacy and comprehension in schools and charities. | consider digital literacy, and expand this investigation to different sectors. |
| Sharman et al., 2024 | Sharman, S., Piper, T., McGrane, E., & Newall, P. W. | 2024 | UK | https://doi.org/10.1080/16066359.2023.2245330 | Empirical Study | **** | Quantitative, frequency analysis of matches shown on ITV during the 2022 Qatar World Cup (N = 30) | Live sports television coverage | Whistle-to-whistle ban | • Whistle-to-Whistle Ban (https://www.gov.uk/government/publications/review-of-the-gambling-act-2005-terms-of-reference-and-call-for-evidence/review-of-the-gambling-act-2005-terms-of-reference-and-call-for-evidence) | 176 gambling-related advertisements were seen, including 20 (11.4%) for Gamble Aware. The following results relate to the 156 advertisements that were from gambling companies or promoting gambling specifically. Across 30 matches, 5.2 (SD = 1.2) advertisements per match were found. There were significantly more shown during matches with home nations compared to not ($p = .013$, $d = 1.24$). The most common were financial inducements (42.3%). Of the 156, 126 were shown pre-match (80.8%), five between kick off and the match end (3.2%) and 25 were shown following the end of the match (16%). 139 contained safer gambling messages (89.1%). | An exception to the whistle-to-whistle ban are lottery products, which were seen during mostly during half-time breaks. It was argued that while the advertisements seen were not in breach of the ban, the most optimum time to advertise would be the pre-match. | An advertising ban not covering all forms of gambling will still allow for exposure. Restrictions could include all types of gambling, across all forms of TV coverage of the match, not just from kick-off to full-time. | To create a dedicated account to record the full broadcasts to ensure everything is captured. It was recommended to not rely on catch-up TV but analyse the live coverage. Future studies could explore advertisements per region and time (catch-up vs live). |
| Smith et al., 2019 | Smith, J., Nairn, A., Rossi, R., Jones, E., & Inskip, C. | 2019 | UK | https://www.bristol.ac.uk/media-library/sites/news/2019/aug/D1261_Horne_DEMOS_Management%20report_2019_web.pdf | Empirical Report | N/A | Mixed-method Context analysis of tweets, manual ( N = 800), and computer aided analysis (N = 888,000). | Twitter based gambling advertising and a sub-focus on how it is consumed by young audiences. | ASA regulations and UK Gambling regulations, CAP regulatory Code; Cap Code 2.1. | • CAP Code (https://www.asa.org.uk/static/c6be0fb9-2c66-4248-ba5b824bf26fd3d3/e8d86fa9-9b16-42db-a6d6ecd93cf8cda0/The-CAP-Code.pdf (Cap Code 2.1; Page 16))<br>• BCAP Code (https://www.asa.org.uk/static/699c12ab-3a81-4175-9a22f8b900997394/6f1bc1a1-6d6c-4e63-9990fc0a4ee766cf/The-BCAP-Code-Gambling.pdf)<br>• UK gambling regulations include: License conditions and code of practice (https://www.gamblingcommission.gov.uk/licensees-and-businesses/lccp/print)<br>• UK Gambling | The analysis of advertising tweets resulted in two gambling ecosystems: prolific accounts related to traditional sports and the growing community of esports. There is a rise in new content, specifically regarding e-sports which are more appealing to young groups. Thousands of children engage with betting content on Twitter, with 28% of those interacting with esports tweets in the UK being underage. Betting advertising on the platform is widespread, often promotes free bets, rarely includes age limits or responsible gambling information, is seldom labelled as advertising, and primarily uses content marketing to build brand awareness and encourage sharing. | The scale and nature of gambling advertising on Twitter raises concerns for regulators, social media platforms, and the gambling industry. While CAP Code 2.1 requires marketing communications to be clearly identifiable as such, the study found no labelled adverts, and the majority of tweets observed were potentially in breach of regulatory standards. | Existing regulations around access to gambling advertising need to be clarified and enforced. Promotions that appeal to vulnerable groups need to be urgently examined. Technology companies should make age verification tools available to all advertisers, enable easy embedding of terms and conditions, and maintain a free, searchable database of gambling adverts. Industry should include explicit references to risk, age restrictions, and responsible gambling in advertising content. Regulators should ensure licensed esports betting operators comply with existing rules, maintain oversight of unlicensed operators, and assess compliance, potential need for new regulations, and the value of education initiatives. | The effects of promotions on young people's gambling behaviours need to be understood. The proliferation of promotions likely to appeal to vulnerable groups, particularly 'free' and 'matched' betting, needs urgent examination. Two areas requiring immediate attention are the use of under 25s in esports advertising and the clarification of the subjective 'particular appeal to children' regulation in the context of the young esports audience. Regulators must ensure they are able to measure and understand the developing landscape of online discussion and respond to new forms of gambling. |

| Study | Authors | Year | Setting | DOI (for studies) URL (for reports) | Record type | MMAT | Study design | Advertising types examined / Marketing channels | Restriction/ policy examined | Restriction/ policy document(s) | Findings/outcomes | Restriction/policy implications | Policy recommendations | Knowledge gaps (future research) |
|---|---|---|---|---|---|---|---|---|---|---|---|---|---|---|
| | | | | | | | | | | Act 2005 (https://www.legislation.gov.uk/ukpga/2005/19/pdfs/ukpga_20050019_en.pdf) | | | | |
| Thomas et al., 2017 | Thomas, S. L., Randle, M., Bestman, A., Pitt, H., Bowe, S. J., Cowlishaw, S., & Daube, M. | 2017 | Australia | https://doi.org/10.1186/s12954-017-0173-y | Empirical Study | **** | Mixed methods, cross-sectional online survey with adolescents and adults (N = 500) aged 16 and over. Also includes qualitative responses with a basic thematic analysis. | Gambling advertising generall, but mainly via television and sponsorships | Proposed ban on gambling advertising during children's viewing hours | • Proposal on ban (https://www.pm.gov.au/media/strong-action-tackle-gambling-harms) | In this study, there was strong support for banning gambling promotions on televised sports (80%) and in sporting venues (77%). The majority of participants agreed with the proposed ban on gambling advertising during children's viewing hours (91.4%). Participants also felt that sporting organisations should take more responsibility when promoting gambling. | Even with a ban on gambling advertising during children's hours, it was highlighted that children would not just stop watching sporting matches at 8.30pm and therefore would still be exposed to gambling promotions. | The current approaches to gambling are out of step with how communities feel about gambling and what the best ways are to protect people, which can include comprehensive restrictions on advertising during sport. | To investigate differences according to age, gender, or gambling characteristics |
| Thomas et al., 2018 | Thomas, S. L., Bestman, A., Pitt, H., Cassidy, R., McCarthy, S., Nyemcsok, C., Cowlishaw, S., & Daube, M. | 2018 | Australia | https://doi.org/10.1186/s12954-018-0254-6 | Empirical Study | **** | Mixed-methods, one-to one interviewer assisted surveys with 11-16 year olds (N = 111). | Gambling advertising when viewing sports-related content on social media. | Mention of the proposed ban on gambling advertising during children's viewing hours (5pm-8:30pm) under the Commercial Television Industry Code of Practice | • Commerical Television Industry Code of Practice (https://www.pm.gov.au/media/strong-action-tackle-gambling-harms) under (https://www.freetv.com.au/wp-content/uploads/2019/07/Free_TV_Commercial_Television_Industry_Code_of_Practice_2018.pdf) | Most (96.4%) of the young people recalled seeing gambling advertising on media platforms and television (91%). When specifically asked about the timing of these, most (67%) reported seeing them before 8:30pm, followed by late evening after 8:30pm (45%), afternoons (23.4%) and mornings (9%). Social media platforms such as YouTube and Instagram were also mentioned. In terms of restrictions, the majority reported that they continued to watch sports after 8:30pm (83.7%) which when the restriction would end. Three quarters agreed that sporting codes should do more to prevent young people from exposure in sports (75.7%) | Participants discussed seeing gambling advertising on social media, which there are no restrictions for. Young people highlighted seeing gambling advertisements early evening before 8:30pm and after 8:30, indicating that the proposed regulations are not enough. | Whistle-to-whistle bans are necessary to limit the exposure to gambling advertising as well as restricting stadium-based advertising. Policymakers should implement measures based on robust findings, as done in other areas of public health. | Future regulations should continue to investigate the current regulatory system. |
| Torrance et al., 2023 | Torrance, J., Heath, C., Andrade, M., & Newall, P. | 2023 | UK | https://doi.org/10.1556/2006.2023.00066 | Empirical Study | ***** | A frequency analysis of logos associated with gambling, cryptocurrency, and financial trading across 10 broadcasts from the 2022/2023 EPL season. | Gambling advertisements during televised football matches | Voluntary EPL front of shirt ban, harm reduction or age restriction warning | • Premier league commitment (https://www.premierleague.com/en/news/3147426) • Mentioned in White Paper (https://www.gov.uk/government/publications/high-stakes-gambling-reform-for-the-digital-age/high-stakes-gambling-reform-for-the-digital-age) | A total of 1,075 individual shirt-front gambling-associated logos were identified representing 6.9% of all individual gambling associated logos and 5.1% of all other relevant logos combined. The most popular location across all relevant logos was pitch-side hoardings (52.3%) via full-coverage dynamic displays, but also included other locations such as shirt sleeves and the stadium structure, highlighting that the front of shirt ban will only apply to approximately 1 in 20 gambling and gambling like logos. Only 3.4% of all relevant brand logos featured harm-reduction or age-restriction warnings. | As the front of shirt voluntary ban would only cover 1 in 20 gambling and gambling-like logos, the ban would be largely ineffective as the logos would still appear in other areas. | N/A | Further research related to logo prevalence and research designs that can uncover potential causal effects on behaviour. |
| Torrance et al., 2026 | Torrance, J., Wright, S., | 2026 | UK | https://doi.org/ | Empirical Study | ***** | Mixed methods, | Financial inducements | UK Gambling Commissions | • Proposed changes to | In the experiment, 269 out of 291 (92.4%) underestimated the | The finding of this study suggest that lowering the | While an outright ban of financial inducements to | A randomised controlled trial in |

| Study | Authors | Year | Setting | DOI (for studies) URL (for reports) | Record type | MMAT | Study design | Advertising types examined / Marketing channels | Restriction/ policy examined | Restriction/ policy document(s) | Findings/outcomes | Restriction/policy implications | Policy recommendations | Knowledge gaps (future research) |
|---|---|---|---|---|---|---|---|---|---|---|---|---|---|---|
| | Newall, P., Crawford, T., Quigley, M., & Dymond, S. | | | 10.1007/ s10899-026-10491-6 | | | online experiment and semantic thematic analysis with adult gamblers (N = 585). | including the wagering requirement needed to withdraw bonus winnings (x10 requirement) | 2026 regulations (a cap on wagering requirements at a multiple of x10). | Licence Conditions and Codes of Practice (LCCP) Social Responsibility (SR) Code 5.1.1 (Rewards and Bonuses) to make the structure and wording clearer (https://www.gamblingcommission.gov.uk/consultation-response/autumn-2023-consultation-proposed-changes-to-lccp-and-rts-socially/proposal-3-proposed-changes-to-lccp-sr-code-5-1-1-to-make-the-structure-and) | inducement and only 16 (5.5%) estimated the requirement correctly. There were significant underestimations overall. However, gambling severity (measured using the PGSI) was not related to the underestimation. When the true requirement was shown, participants found this significantly less attractive compared to when it was not shown. It was suggested that higher-risk bettors may be less deterred by cost disclosures. In the qualitative element, participants described financial inducements as manipulative and predatory, akin to a scam or 'con', and in need of better regulation. | wagering multiples (per the Gambling Commission's 2026 cap) is insufficient to ensure fully informed choices. | new customers may be unlikely, applying disclosure requirements that are used for mortgages, credit cards, and other financial products would be a good first step. | partnership with gambling operators using player account data would help to examine actual inducement behaviour. Replication across different recruitment methods and countries would also help with generalisability. To assess comprehension in both conditions to see which examples improve comprehension. Employing a choice paradigm task with multiple inducements with varying wagering requirements and to determine which elements of what combinations of inducements present the greatest barriers. |
| Ukhova et al., 2024 | Ukhova, D., Marionneau, V., Nikkinen, J., & Wardle, H. | 2024 | Global | https://doi.org/10.1016/S2468-2667(23)00221-9 | Empirical Study | ***** | A review of legislative and regulatory changes between 2018 and 2021 | Gambling advertising and marketing globally | Included regulation from 48 countries. Restrictions mainly covered advertising to vulnerable groups such as underage people, self-excluded, and at-risk players. | See Table S1 in Appendix | Since 2018, 21 of 48 (43.8%) of European jurisdictions have introduced some limitations on gambling advertising and marketing, compared with 3 of 27 (11.1%) in Asia. However, Asia also contained a large number of countries that do not permit gambling. There was a ban on all sports betting advertising in Albania but was later lifted, while Argentina simply had a statement commenting that advertising with authorisation from the regulator was not permitted. Most restrictions were focused on vulnerable groups such as underage people (15 of 21; 71%). Some, but very few, had measures aimed to reduce exposure to gambling (6 of 21; 29%). | The review noted that most jurisdictions had restrictions that were focussed on protecting vulnerable individuals from gambling advertising and marketing, rather than population wide. | Gambling harm policies (including advertising) should encompass the wider population, rather than focussing on select individuals. | Future research could reapply this approach to other jurisdictions such as the USA or India and include newly liberalised countries. Qualitative assessment of advertisements or duty of care could also be conducted to assess whether they are protecting the wider public. |
| Wheaton et al., 2025 | Wheaton, J., Rossi, R., Moxey, M., Tozzi, E., & Moradipour, S. | 2025 | Canada | https://doi.org/10.1080/14459795.2024.2400356 | Empirical Study | ***** | A frequency analysis of gambling advertisements during two NBA and five NHL matches; a content analysis of Twitter/X advertisements. A total of 4,119 | Gambling advertising during televised sports matches and on social media platforms. | AGCO restrictions: (1) target high-risk, underage or self-excluded persons; (2) mislead players or misrepresent gambling; (3) carry inducements, except for on an operator's | • AGCO restrictions https://www.agco.ca/en/lottery-and-gaming/responsible-gambling/marketing-and-advertising | Age restriction and harm reduction messages were not widely prevalent with 2.6% of references containing harm reduction and 2.8% constraining any age restriction message. These were mostly found during commercial break advertisements and other formats not on the playing surface, court surface, or rink side hoardings. On social media platforms, while 35.7% of conventional advertisements | The research highlighted the lack of harm reduction and age warning restrictions in the advertisements observed. The authors expressed that explicit guidelines are needed around content marketing, which are currently missing. | Existing regulations could be developed to include regulate advertisements that appear most frequently on the playing surface to reduce exposure to underage audiences. The authors also recommended any future regulations being led by policymakers to avoid commercial interests. | To: explore the normalisation of gambling in North American sports leagues through increased advertisements, analyse an equal number of NHL and NBA matches and include longitudinal research, explore the reach of content posted by Canada-based |

| Study | Authors | Year | Setting | DOI (for studies) URL (for reports) | Record type | MMAT | Study design | Advertising types examined / Marketing channels | Restriction/ policy examined | Restriction/ policy document(s) | Findings/outcomes | Restriction/policy implications | Policy recommendations | Knowledge gaps (future research) |
|---|---|---|---|---|---|---|---|---|---|---|---|---|---|---|
| | | | | | | | gambling-related messages across all platforms. | | site or through direct marketing; or (4) describe inducements – where permitted – as risk-free or free, and make the terms and conditions clearly available. Additionally (5), players should be given access to an opt-in process where they can consent to receive direct marketing. Canadian Ad Standards (self-regulated) | | contained harm reduction and age restrictions, these were only present in 1.4% of content marketing advertisements. Only four (1.4%) of content marketing advertisements were clearly labelled as advertising. | | | operators on social media platforms. |
| Wilson et al., 2024 | Wilson, J., Rossi, R., Bransden, N., Amos, M., & Sakis, P. | 2024 | Italy, Spain, Germany, Netherlands, Belgium, Australia and comparing to GB | https://www.gambleaware.org/media/bjqev1hx/drivers-of-gambling-marketing-restrictions-an-international-comparison-v3.pdf | Technical Report | N/A | A document review and a series of semi-structured interviews and email exchanges | Sports sponsorships, commercials during sports broadcasts, public posters, online advertising, TV and radio advertising, social media advertising, targeted advertisements. | Gambling Act 2005, CAP and ASA including voluntary whistle to whistle and before 21:00 bans. Dignity Decree 2019 (Italy). Royal Decree 2020 (Spain). Gambling Act 2021 (Germany), Gambling Act 1999 (Belgium) and 2018 Royal Decree 2023 (Belgium), ACMA (Australia). | • Gambling Act 2005 (https://www.legislation.gov.uk/ukpga/2005/19/contents)<br>• Italy Dignity Decree (https://www.normattiva.it/uri-res/N2Ls?urn:nir:stato:decreto.legge:2018-07-12;87); Spain Royal Decree (https://www.boe.es/buscar/pdf/2020/BOE-A-2020-13495-consolidado.pdf); Germany (https://www.gesetze-bayern.de/Content/Document/StVGlueStV2021/true)<br>• Belgium 1999 (https://www.gamingcommission.be/fr/legislation/loi-sur-les-jeux-de-hasard-du-7-mai-1999)<br>• Belgium 2023 (https://www.ejustice.just.fgov.be/cgi_loi/article.pl?language=fr&dt | GB has the highest level of research on gambling harm and marketing but relatively few advertising restrictions compared with other jurisdictions. In contrast, Italy has introduced a full ban on gambling marketing, although this has been challenged by industry and football stakeholders due to concerns about job losses. Spain has implemented near-total restrictions, including bans on sponsorship, strict limits on broadcast and online advertising, removal of public figures in ads, restrictions on bonuses, tighter controls on promotional offers, and limits on access to free-to-play content.<br>Germany allows some advertising but restricts it during sports broadcasts, while the Netherlands permits most advertising except online and targeted forms, though additional restrictions on high-risk games have been proposed. Belgium has broadly banned gambling marketing apart from sports sponsorships, with limited exceptions (e.g. B2B advertising and some foreign media). In Australia, gambling advertising remains largely permitted but is subject to time-based and live-sport restrictions that vary by state. | Where restrictions have been introduced, the increased normalisation of gambling as a leisurely activity through marketing was challenged by third sectors (Italy) or politicians. When restrictions are implemented, there are debates related to the rise of the black market. In Spain and Netherlands, there are efforts to reverse restrictions. GB is the only country that relies on self regulation and the gambling industry | Primary legislation is required over self regulation in GB. In Australia, a total ban on gambling advertising was discussed. | N/A |

| Study | Authors | Year | Setting | DOI (for studies) URL (for reports) | Record type | MMAT | Study design | Advertising types examined / Marketing channels | Restriction/ policy examined | Restriction/ policy document(s) | Findings/outcomes | Restriction/policy implications | Policy recommendations | Knowledge gaps (future research) |
|---|---|---|---|---|---|---|---|---|---|---|---|---|---|---|
| | | | | | | | | | | =KONINKLIJK+BESLUIT&nl=n&text1=kansspel&choix1=en&trier=afkondiging&lg_txt=f&type=&sort=&numac_search=2023030509&cn_search=&caller=list&&view_numac=2023030509dx2023030509fx2023030509nx2024021809nl) • Australia (https://www.acma.gov.au/rules-gambling-ads) | | | | |
| Worters et al., 2025 | Worters, T., McKerchar, C., Watkins, L., Gage, R., & Signal., L. | 2025 | New Zealand | https://doi.org/10.1016/j.socscimed.2025.118055 | Empirical Study | **** | Quantitative, cross-sectional observation with Year 8 children aged 11-13 years of age (N = 168) across 16 schools using automated wearable cameras. | Unhealthy commodity (UHC) marketing, including gambling. | Gambling Act 2003 (NZ). Self-regulation of UHC marketing via the ASA. | •Gambling Act 2003 (NZ) (https://www.legislation.govt.nz/act/public/2003/51/en/latest/#DLM207497) | A small amount of children's marketing exposure came from gambling (M = 2.1 daily exposures). No gambling marketing found on product packaging but was found on signs (49.9%). Children were exposed to gambling marketing over a diverse range of settings. Only four gambling brands were found to have an exposure rate of more than 0.1 a day. It was found that nearly all (97.4%) the gambling brands found were owned by only two state bodies (Lotto NZ, NZRB). | It was highlighted that statutory restrictions are the most effective approach for protecting children from UHC marketing. Those commodities that were self-regulated (including gambling) were encountered more by children. Thus, self-regulation is largely ineffective. | As most of the gambling marketing in this study was state owned, the government should direct statutory bodies to reduce gambling marketing. | Future research could include real-time screen capture on the devices that children use and monitoring of websites and social media. Māori populations and other indigenous communities could also be included in future studies. |
| Xiao & Deery, 2025 | Xiao, L. Y., & Deery, C. | 2025 | Netherlands | https://osf.io/preprints/osf/62pmw_v1 | Pre-print - Empirical Study | ***** | Quantitative, observation of gambling advertisements (N = 277) targeting 18-23 year olds using the Meta Ad Library | Gambling advertising generally | Besluit werving, reclame en verslavingspreventie kansspelen van 7 mei 2013 (Decree on Recruitment, Advertising and Addiction Prevention of 7 May 2013) | • Decree on Recruitment, Advertising and Addiction Prevention (https://wetten.overheid.nl/BWBR0033412/2021-10-01) | 16 of 220 online gambling licensee ads (7.3%) published by 3 accounts; 14 of 47 (29.8%) non-online gambling licensee ads published four accounts; and 1 of 10 advance-published ads (10%) targeted users within the prohibited range in breach of the Decree. A test of proportions also revealed that online gambling advertisements were more likely to comply than offline gambling advertisements (z = 4.45, p <.001). Overall, this means that 31 ads were in breach of the Decree. | There was a high compliance rate (92.7%) among ads published by online gambling licences, but a lower compliance rate (70.2%) for off gambling licensee ads. However, there was a minority pf gambling ads that were illegally targeting young adults in Netherlands. | Broadly, stricter enforcement by regulators was recommended. Meta and other platforms should allow advertisers to set minimum target ages by default or apply industry-specific rules in the relevant country. | Further research of demographic groups including women, older people, migrants, and ethnic minorities. |

* The original papers (Hing et al., 2016; Johns et al., 2017) used the term ‘problem gambler’ at these points. We have substituted person-first wording to avoid stigmatising language, without altering the meaning of the findings as reported by those authors.
